# Integrating Multi-Omics and Machine Learning to Reveal a Prognostic Model for Prostate Cancer Metastatic Recurrence Associated with Epithelial–Mesenchymal Transition Features

**DOI:** 10.3390/genes17091015

**Published:** 2026-08-27

**Authors:** Xueqian Zhang, Wei Zhang, Zheng Wang, Xinyang Shi, Chenghao Zhang, Yan Gao, Yiheng Deng, Tianyu Shen, Ziyan An, Weijun Fu

**Affiliations:** 1 School of Medicine, Nankai University, 94 Weijin Road, Tianjin 300071, China; 2 Department of Urology, PLA General Hospital, Beijing 100039, China; 3 School of Medical Technology, Hebei Medical University, Shijiazhuang 050031, China; 4 Department of Urology, Beijing Shijitan Hospital, Capital Medical University, Beijing 100038, China

**Keywords:** prostate cancer, epithelial–mesenchymal transition, tumor microenvironment, machine learning, multi-omics analysis

## Abstract

**Background**: Prostate cancer (PCa) is a leading cause of cancer-related mortality worldwide, highlighting the need for improved prognostic tools. The integration of artificial intelligence (AI) and machine learning (ML) with multi-omics data offers new opportunities for biomarker discovery and risk stratification. **Methods**: We integrated bulk transcriptomic data from GSE116918 (training, *n* = 248) and three cross-cohort consistency evaluation cohorts (TCGA-PRAD, GSE70769, GSE46602), focusing on 1087 epithelial–mesenchymal transition (EMT)-associated genes. Using consensus clustering, weighted gene co-expression network analysis (WGCNA), and 91 machine learning algorithm combinations (including Random Forest, Lasso, and CoxBoost), we constructed a prognostic signature. SHAP analysis was used for model interpretability. Single-cell RNA sequencing (scRNA-seq, GSE268307, 10,672 cells) and spatial transcriptomics (10× Genomics Visium FFPE) provided hypothesis-generating evidence; spatial analysis was based on one tissue section. **Results**: A three-gene signature (INHBA, FAP, ITGBL1) effectively stratified patients into high- and low-risk groups, with the high-risk group showing significantly worse metastasis-free survival (HR = 1.61, 95% CI: 1.39–1.87; 4-year AUC = 0.93 in the training cohort; external AUCs ranged from 0.62 to 0.77). CytoTRACE inferred high differentiation potential of COMP+ fibroblasts, and Monocle3 inferred a transcriptional transition from COMP+ toward NELL2+ fibroblasts. BayesPrism deconvolution suggested that high inferred COMP+ fibroblast abundance was associated with poor prognosis and advanced T stage. NicheNet analysis prioritized BMP7 as a key upstream ligand, with downstream targets enriched in TGF-β signaling and stem cell pluripotency pathways. **Conclusions**: This study presents a machine learning-based multi-omics framework for prostate cancer risk stratification. The three-gene signature provides a new exploratory prognostic model while inferring a COMP+ to NELL2+ transcriptional transition. These findings may inform future hypothesis-driven studies of treatment sensitivity, pending experimental validation, and demonstrate the value of AI-driven multi-omics integration for precision oncology.

## 1. Introduction

Prostate cancer stands as the second most common male malignancy globally and the fifth leading cause of cancer-related death, presenting a major challenge to public health [1]. Its incidence and prevalence vary sharply by geography and race—rates are far higher in Europe, North America, and South Africa than in Asia [2]. The disease primarily affects older men, with around 60% of diagnoses occurring in those over 65 [3]. Age, family history, and genetic susceptibility thus play pivotal roles in prostate cancer development and progression [4]. To date, genome-wide association studies (GWAS) have identified nearly 270 gene loci containing hundreds of single-nucleotide polymorphisms (SNPs) linked to prostate cancer susceptibility [5], and recent large-scale GWAS have added over 170 common genetic variants associated with disease risk [6]. Current treatments include local therapies (surgical resection, radiation), androgen deprivation therapy (ADT), and chemotherapy [7]. Biochemical relapse (BCR)—marked by rising prostate-specific antigen (PSA) levels post-initial treatment—signals potential local recurrence or distant metastasis [8], highlighting the need for reliable prognostic models to improve survival prediction and stratify metastatic risk in prostate cancer patients. Epithelial–mesenchymal transition (EMT) is a dynamic cellular reprogramming process where epithelial cells reduce epithelial marker expression, adopt mesenchymal traits, develop fibroblast-like morphology, and gain enhanced migratory and invasive capabilities [9]. Recognized as a driver of tumor invasion, metastasis [9], accelerated proliferation, immune escape, and resistance to senescence and apoptosis [10], EMT disrupts tissue structure by altering cell–cell and cell–matrix interactions—allowing cells to detach from the basement membrane and activate mesenchymal transcriptional programs. Importantly, EMT influences tumor progression, chemotherapy response, patient prognosis, and progression-free survival [11], making systematic analysis of EMT-related gene networks valuable for prognostic stratification, clinical decision-making, survival assessment, and novel therapeutic target discovery. While EMT-related genes have been studied for prognostic value in various cancers, a comprehensive EMT-based risk model for prostate cancer and its clinical translation remain incomplete. To address this gap, we selected 117 prognosis-associated EMT genes to stratify the GSE116918 cohort into molecular subtypes. Integrating limma differential expression analysis and WGCNA, we identified key genes critical to prostate cancer progression. We then applied 91 machine learning algorithms combined with SHAP (SHapley Additive exPlanations) analysis to screen high-value prognostic biomarkers, construct a multi-gene risk model, and analyze its associations with tumor immune infiltration, drug sensitivity, and tumor mutational burden (TMB). Single-cell and spatial transcriptomics further clarified the spatial expression dynamics of the model within the prostate tumor microenvironment. This study provides computational hypotheses that may inform future treatment-related studies.

## 2. Materials and Methods

### 2.1. Data Acquisition and Processing

Training cohort: GSE116918 comprised 248 prostate cancer patients treated with radical radiotherapy. Of these, 22 patients had metastatic recurrence events and 226 patients did not (MFS endpoint used for model training); BCR events were 56, with 192 patients without BCR. The final three-gene model therefore had an events-per-variable ratio of 22/3 = 7.33. Given the limited number of events relative to predictors, the model is regarded as exploratory and at risk of overfitting. The expression matrix of GSE116918 was annotated based on GPL25318 and ENSG identifiers, and converted to official human gene symbols; duplicate gene symbols were processed by averaging their expression values.

Validation cohorts: TCGA-PRAD was downloaded from the UCSC Xena database (https://xena.ucsc.edu/ (accessed on 20 June 2025)). Samples with missing PFS information were removed. PFS was defined as the occurrence of a new tumor event, including disease progression, local recurrence, distant metastasis, or a new primary tumor at any site, or death from any cause; cases with a new tumor event of unspecified type were retained. GSE70769 comprised tumor tissue from robotic radical prostatectomy, with 45 BCR events and 48 patients without BCR. GSE46602 comprised laser-microdissected prostate tumor tissue, with 22 BCR events and 14 patients without BCR. Normal tissue samples were excluded from the TCGA-PRAD dataset, and the FPKM expression values were normalized by log_2_(x + 1) transformation with duplicate genes averaged for correction. GSE70769 and GSE46602 datasets were, respectively, annotated according to their corresponding detection platforms, GPL10558 and GPL570. A total of 1294 epithelial–mesenchymal transition (EMT)-related genes were collected by merging and deduplicating gene sets from the MSigDB (https://www.gsea-msigdb.org/gsea/msigdb (accessed on 20 June 2025)) and dbEMT (https://bioinfo-minzhao.org/dbemt/dbemt1/index.html (accessed on 20 June 2025)) databases [12], among which 1087 genes were successfully matched in the GSE116918 training cohort and included in subsequent analyses. The EMT-related genes from different databases are stored in Table S1.

### 2.2. Univariate and Multivariate Cox Regression Analyses

Cox regression analyses were performed using R’s “survival” package, integrating clinical follow-up data (recurrence time, with last follow-up time for non-recurrence cases), recurrence status, and EMT-related gene expression levels. Univariate models assessed individual gene prognostic value, while multivariate models evaluated gene sets. Hazard ratios (HR) and 95% confidence intervals (CI) were calculated for each gene, and the Wald test determined regression coefficient significance (two-tailed *p* < 0.05).

### 2.3. Kaplan–Meier Curve Generation

We used the maxstat R package (version 0.7-25) to determine optimal expression thresholds for EMT-related genes, splitting patients into high- and low-expression groups. Kaplan–Meier survival curves were generated with the “survival” package’s survfit function to compare survival between groups, and the Wald test verified significance (*p* < 0.05).

### 2.4. Consensus Clustering

Consensus clustering was conducted with R’s ConsensusClusterPlus package to explore relationships between EMT-related gene expression profiles and prostate cancer molecular subtypes [13]. We used a distance metric based on the 1-Pearson correlation coefficient and the partition around the centroid (PAM) algorithm. To ensure result stability, 80% of samples were randomly resampled for 10 iterations. The optimal number of clusters was determined by analyzing the empirical cumulative distribution function (CDF) curve and relative changes in the area under the CDF curve.

### 2.5. Principal Component Analysis (PCA)

The gene expression data were z-score-normalized before analysis. PCA was performed using the prcomp function in the “stats” package (version 4.5.2) to reduce dimensionality and separate samples, making the results more visual.

### 2.6. Gene Set Variation Analysis (GSVA)

The “gsva” package was used to calculate enrichment scores for EMT-related gene sets and other pathways relevant to prostate cancer, such as the androgen receptor network, across individual samples. Differences in pathway activity between clusters C1 and C2 were compared using the Wilcoxon rank-sum test. Pathway definitions were obtained from the PathCards (https://pathcards.genecards.org/ (accessed on 10 July 2025)) and MSigDB databases [12].

### 2.7. Differential Expression Analysis with Limma

R’s limma package (version 3.66.0) identified differentially expressed genes (DEGs) between C1 and C2 clusters [14]. Linear models were built with lmFit, empirical Bayesian correction applied via eBayes, and corrected t-statistics calculated. DEGs were defined as those with |log_2_(fold change)| > 1.5 and false discovery rate (FDR) < 0.05—yielding 186 DEGs (146 upregulated, 40 downregulated in C2 relative to C1).

### 2.8. Weighted Gene Co-Expression Network Analysis (WGCNA)

Standardized gene expression data were filtered to exclude genes with zero variance across samples, and outliers were removed using the WGCNA package’s goodSamplesGenes function. A soft threshold power (β) of 10 was chosen to ensure a scale-free co-expression network topology. We calculated the Topological Overlap Matrix (TOM) and performed hierarchical clustering based on TOM dissimilarity to identify gene modules (minimum module size = 30). Modules with feature gene dissimilarity < 0.25 were merged, resulting in 10 distinct co-expression modules (unassigned genes formed the gray module).

### 2.9. Protein–Protein Interaction (PPI) Network Construction

We predicted the protein–protein interaction relationships among the encoded products of different hub genes using the STRING online database (https://cn.string-db.org/ (accessed on 10 June 2025)). The obtained interaction network was visualized with bioinformatics tools, and we further analyzed the network topology to screen out core protein–protein interaction pairs and key regulatory nodes. All parameters are set to default, and the species selection is set to “homo”.

### 2.10. Gene Ontology (GO) and Kyoto Encyclopedia of Genes and Genomes (KEGG) Enrichment Analyses

GO enrichment analysis was conducted using the clusterProfiler package with annotations from org.Hs.eg.db as background. KEGG pathway analysis was performed using gene sets from MSigDB as the reference [12]. Significantly enriched terms were identified with a minimum gene set size of 5, a maximum of 5000, *p* < 0.05, and FDR < 0.05.

### 2.11. Prognostic Model Construction

We applied a combination of 91 machine learning algorithms to 28 prognostic-related genes (which were identified and screened by univariate Cox regression). These algorithm combinations were derived from 9 basic algorithms, including random survival forest, elastic network, Lasso regression, ridge regression, stepwise Cox regression, CoxBoost regression, plsRcox regression, SuperPC regression, and survival support vector machine (survival-SVM). In the GSE116918 cohort, the model was trained using leave-one-out cross-validation (LOOCV). The model was used for cross-cohort consistency evaluation during model development (TCGA-PRAD, GSE46602, GSE70769) using the “Mime1” package [15]. Finally, the model with the highest average Harrell consistency index (C index) was selected as the optimal model across all cohorts.

### 2.12. SHapley Additive exPlanations (SHAP) Analysis

SHAP values were computed using the “fastshap” package to interpret the contribution of each feature to the optimal model. The “shapviz”, “ggplot2”, and “patchwork” packages were used for visualization [16].

### 2.13. Nomogram Construction and Validation

This study used the “rms” package to construct a nomogram containing age, three gene signatures, and PSA levels. This nomogram can be used to predict the probability of 4-year, 6-year, and 8-year metastasis-free survival (MFS) in prostate cancer patients. Calibration curves were plotted to assess the consistency between the predicted results and observed results. To evaluate the clinical utility of different variable combinations, we constructed three Cox proportional hazards models: a full model including age, risk score, and PSA; an age-only model; and a PSA-only model. Individualized 4-year (1460-day) event risk probabilities were estimated using the survival function for each patient. Decision curve analysis (DCA) was then performed to compare the net benefit of the three models across a range of threshold probabilities, using the “treat all” and “treat none” strategies as references, thereby assessing the clinical usefulness of each model.

### 2.14. Transcription Factor (TF)–microRNA (miRNA)–Gene Network Analysis

We constructed a TF-miRNA-gene regulatory network involving 28 prognostic-related genes using the NetworkAnalyst online analysis platform (https://www.networkanalyst.ca/ (accessed on 12 August 2025)) [17,18,19,20,21]. The regulatory relationship data of TFs and miRNAs targeting the above genes were retrieved from miRTarBase v9.0 and TRRUST databases. The screening criteria for network nodes were set as follows: specific to GTEx prostate tissue, node degree ≥ 1, and betweenness centrality ≥ 2.

### 2.15. Receiver Operating Characteristic (ROC) Curve Analysis

Time-dependent ROC curve analysis was conducted with R’s “pROC” package to assess prognostic gene prediction accuracy at different time points. Area under the curve (AUC) and 95% confidence intervals (CI) were calculated to quantify the signature’s predictive performance.

### 2.16. Immune Infiltration Analysis

Infiltration levels of 28 immune cell types were quantified using single-sample gene set enrichment analysis (ssGSEA) via the “GSVA” package [22]. Additional algorithms, including CIBERSORT and ESTIMATE, were also applied [23,24]. Differences in immune scores between risk groups were compared using the Wilcoxon rank-sum test.

### 2.17. Tumor Mutation Burden Analysis

Somatic mutation data of TCGA-PRAD samples were downloaded from the Genomic Data Commons (GDC) portal (https://portal.gdc.cancer.gov/ (accessed on 20 June 2025)). We categorized nonsynonymous mutations as functional variants, while silent mutations and non-coding region mutations were defined as wild-type variants. Mutation profiles of the samples were visualized using the “maftools” package in R, and we further compared the differences in mutation spectra and mutation frequencies between different risk groups of prostate cancer patients.

### 2.18. Drug Sensitivity Prediction

The “oncoPredict” package was used to estimate chemotherapeutic sensitivity based on gene expression profiles [25]. Differential drug response between risk subgroups was assessed using the Wilcoxon rank-sum test.

### 2.19. Single-Cell RNA Sequencing Analysis

The GSE268307 single-cell RNA sequencing dataset (10× genomics single-cell RNA sequencing analysis on human surgical specimens of 2 localized and 2 metastatic hormone-naïve prostate cancer patients, with 4 prostate gland biopsies in total) was processed via the Seurat pipeline. Cells were filtered by three criteria: number of detected genes (nFeature_RNA) < 10,000, total UMIs (nCount_RNA) < 100,000, and mitochondrial gene expression ratio < 20%. Batch effects between samples were eliminated using the soft k-means clustering algorithm in the Harmony package [26]. Dimensionality reduction and cell clustering were performed with UMAP, and cell type annotation was completed by combining the HumanPrimaryCellAtlasData reference dataset (celldex package) and the SingleR annotation tool [27]. Finally, we conducted manual annotation for correction: ACTA2 for smooth muscle cells, KRT5 and EPCAM for epithelial cells, PECAM1 and VWF for endothelial cells, CD3G and CD8A for T cells, S100A8 and LYZ for monocytes, KLK3 and AR for cancer cells, TPSAB1 for mast cells, and IGHG4 for B cells, SOX10 and S100B for neurons.

### 2.20. Spatial Transcriptomics Data Processing

Spatial transcriptomics data of human prostate invasive adenocarcinoma (FFPE sample) was obtained from the 10× Genomics public database (https://www.10xgenomics.com/datasets (accessed on 22 July 2025)). All data processing and normalization were performed in Seurat software (version 5.5.0) using the sctransform method for gene expression normalization and variance stabilization (https://satijalab.org/seurat/articles/spatial_vignette.html (accessed on 22 July 2025)).

### 2.21. Spatial Cell Type Deconvolution Based on RCTD

Using single-cell RNA sequencing reference data [28], we estimated spatial transcriptomics cell type composition at each spot via the robust cell type decomposition (RCTD) method. RCTD deconvolved relative cell type abundance across tissue regions for quantitative spatial composition analysis. Deconvolution was performed with the create.RCTD and run.RCTD functions (spacexr package), and results were visualized using the “STdeconvolve” package.

### 2.22. Intercellular Communication Analysis

Potential intercellular communication networks in the prostate tumor microenvironment were inferred by single-cell RNA sequencing data, using the CellChat and FNN packages [29]. By analyzing the expression patterns of ligand–receptor gene pairs, the key intercellular communication pathways in single-cell omics were identified. Network centrality analysis was used to identify core sender and receiver regions in critical tumor progression-related signaling pathways.

### 2.23. Pseudotemporal Ranking and Trajectory Inference

Pseudoyemporal ranking analysis reconstructed cell differentiation trajectories in the tumor microenvironment. First, the CytoTRACE2 R package quantified individual cell differentiation potential, with cells showing the highest potential designated as the starting population. The monocle3 R package then ranked cells along a pseudotimeline to infer dynamic cell evolution from less to more differentiated states and identify key lineage trajectory branching points.

### 2.24. Intercellular Communication Analysis Based on NicheNet

The “nichenetr” R package inferred signaling molecules and regulatory pathways mediating communication from fibroblast subsets to cancer cell in the prostate tumor microenvironment. COMP-positive fibroblasts were defined as signal senders, and cancer cell were defined as receivers. To identify metastasis-associated signaling pathways, single-cell RNA sequencing data from prostate cancer patients without lymph node metastasis served as a control. This comparative analysis prioritized key ligand–receptor pairs and predicted downstream target genes specifically regulated in the metastatic microenvironment.

### 2.25. Cell Type Deconvolution of Bulk Transcriptomic Data

For the deconvolution work of the large-scale RNA sequencing data GSE116918, we adopted the “BayesPrism” method based on the “BayesPrism” R package [30]. The single-cell RNA sequencing dataset with detailed annotations was used as the reference map. We filtered out the abnormal outlier genes in the single-cell data, selected the “protein_coding” with the highest correlation, and finally extracted the posterior average value of the cell type fraction θ. This Bayesian method is used to robustly estimate the abundance of cell types and the cell type-specific gene expression patterns from the overall transcriptome of tumor samples, thereby conducting high-resolution interpretation of the overall data through our single-cell discovery results.

### 2.26. Statistical Analysis

All statistical analyses were performed in R software (version 4.5.2). Continuous variable comparisons used the Wilcoxon rank-sum test (two groups) or Kruskal–Wallis test (multiple groups). Correlation analysis employed Pearson or Spearman correlation coefficients, depending on data normality. The Benjamini–Hochberg correction was applied to the initial univariate Cox screening and limma differential analysis. Descriptive or model-based analyses, including clustering, trajectory inference, CellChat, and NicheNet, did not undergo formal hypothesis testing and were therefore presented in descriptive or predictive computational form. For analyses that could not provide confidence intervals or effect sizes, it was clearly indicated that this was a limitation. A two-tailed *p* < 0.05 was considered statistically significant.

## 3. Results

### 3.1. Schematic Overview of the Analytical Workflow

**Figure 1:**
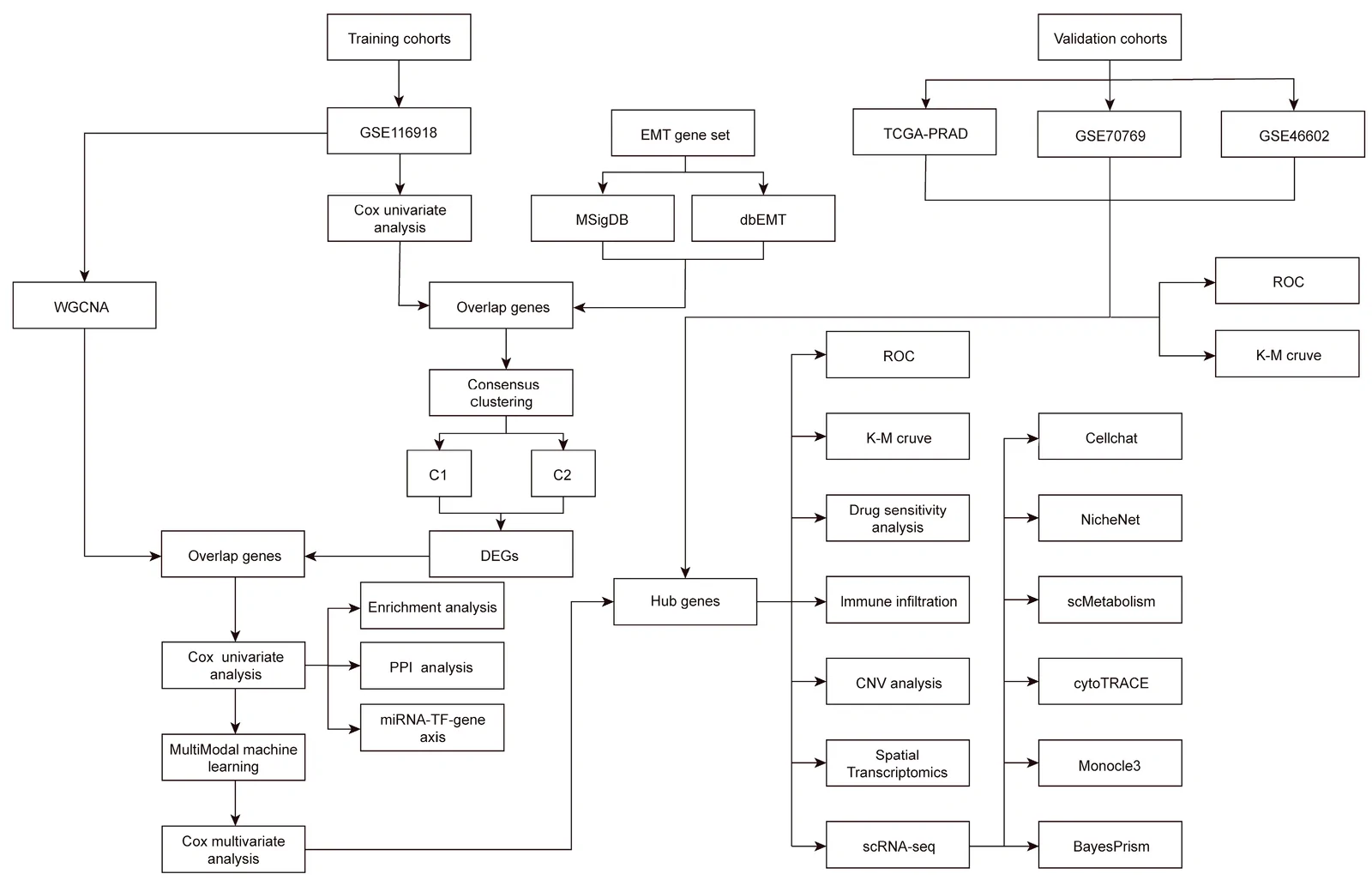
Schematic overview of the analytical workflow. Schematic overview illustrating the integrative bioinformatic and experimental pipeline, from EMT gene screening and prognostic model construction to validation through single-cell and spatial transcriptomic analyses.

The overall design and multi-step bioinformatic strategy employed in this study are summarized in Figure 1. (A) Using the training cohort (GSE116918), a univariate Cox regression model was applied to an EMT gene set compiled from MSigDB and dbEMT to identify prognosis-associated genes. (B) Consensus clustering stratified patients into two distinct subtypes (C1 and C2). DEGs were identified between clusters. (C) WGCNA was performed to identify gene modules significantly correlated with the aggressive subtype (C2). Overlapping genes between DEGs and key module genes were subjected to PPI network construction, enrichment analyses, and regulatory network (miRNA-TF-gene) analysis. (D) Multimodal machine learning algorithms were applied to select hub genes and construct a prognostic signature, which was subsequently validated in three independent cohorts (TCGA-PRAD, GSE70769, GSE46602). (E) The final model was evaluated through survival analysis (Kaplan–Meier curves), ROC curves, immune infiltration profiling, drug sensitivity prediction, and copy number variation (CNV) analysis. (F) Through spatial transcriptomics and single-cell RNA sequencing (scRNA-seq) techniques, the expression and functional roles of key genes in the tumor microenvironment were computationally explored. High-expression hub genes in COMP+ fibroblasts were identified, possessing high differentiation potential. CytoTRACE and Monocle3 inferred a transcriptional transition from COMP+ toward NELL2+ fibroblasts and predicted BMP signaling toward cancer cells. Preliminary validation through deconvolution also suggested an association of COMP+ fibroblasts in clinical data phenotypes.

**Figure 2:**
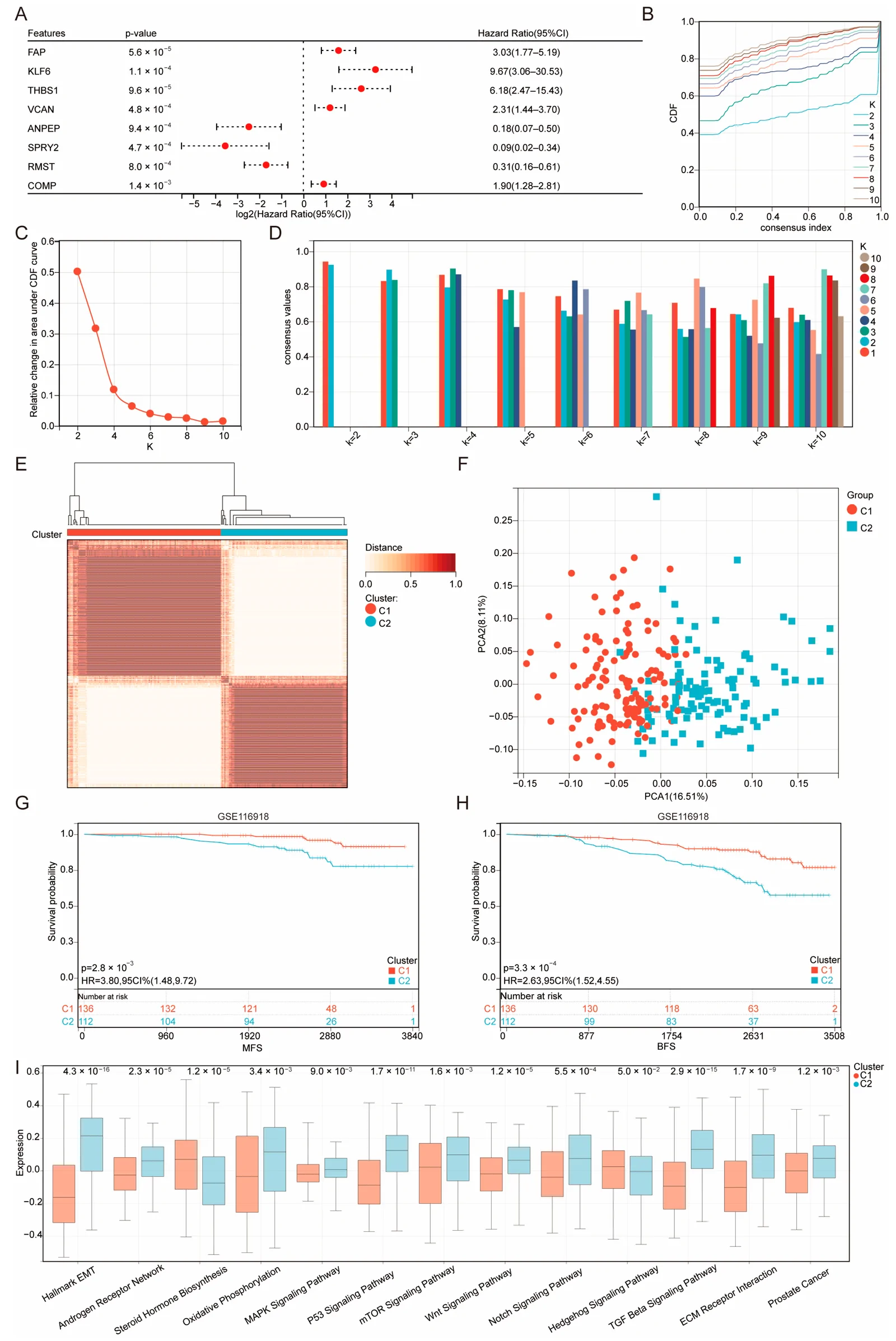
EMT-related gene clustering identifies two distinct prognostic subgroups in the training cohort. (**A**) Forest plot of the top 8 most significant EMT-related genes associated with overall survival from univariate Cox regression analysis. (**B**) CDF curves for consensus clustering under different k values. (**C**) Relative change in the area under the CDF curve for determining the optimal cluster number (k). (**D**) Consensus score across predefined k values, with k = 2 showing optimal stability. (**E**) Heatmap of the consensus matrix for k = 2, demonstrating clear segregation into two clusters (C1 and C2). (**F**) Principal component analysis (PCA) plot validating the distinct transcriptomic profiles of clusters C1 and C2. (**G**,**H**) Kaplan–Meier curves comparing (**G**) MFS and (**H**) BFS between the two clusters. (**I**) Box plots depicting the activation scores of hallmark cancer-related signaling pathways in clusters C1 and C2.

We collected 1294 EMT-related genes from dbEMT and MSigDB databases and performed univariate Cox regression to evaluate their prostate cancer prognostic value. Applying a *p* < 0.05 significance threshold, 117 prognostic EMT-related genes were selected for further analysis. The eight most significant genes are presented in a forest plot (Figure 2A) and corresponding survival curves (Figure S1A–H, Table S2). The consensus clustering of the GSE116918 training cohort, based on these 117 genes’ expression profiles, suggested the need to divide the cohort into two subgroups—evident from the empirical cumulative distribution function (CDF) curve (Figure 2B), relative changes in the area under the CDF curve (Figure 2C), and cluster consensus evaluation (Figure 2D), which showed optimal stability at K = 2. A consensus matrix heatmap further confirmed the two-cluster structure’s robustness (Figure 2E). Principal component analysis (PCA) clearly separated Cluster 1 (C1) and Cluster 2 (C2) (Figure 2F). Kaplan–Meier survival analysis revealed significantly shorter biochemical recurrence-free survival (BFS) and metastasis-free survival (MFS) in C2 patients compared to C1 (Figure 2G,H). KEGG pathway enrichment analysis showed C2 had enhanced activity in EMT, androgen receptor signaling, and multiple tumor-related pathways (oxidative phosphorylation, MAPK, p53, Wnt, mTOR, Notch, TGF-beta) (Figure 2I).

Limma package comparisons between C2 and C1 identified 186 DEGs (146 upregulated, 40 downregulated in C2), visualized in a volcano plot (Figure S2A) and heatmap of the top 30 up/downregulated genes (Figure S2B). WGCNA with a soft threshold power β = 10 (ensuring scale-free topology; Figure S2C,D) identified 10 gene co-expression modules. Sample and gene clustering dendrograms are shown in Figure S2E,F. Module-trait correlation heatmaps (Figure S2G,H) revealed strong links between the black and red modules and the C2 aggressive phenotype, with scatter plots quantifying module eigengene-cluster assignment relationships (Figure S2I–L).

### 3.2. Prognostic Hub Gene Selection and Functional Annotation

**Figure 3:**
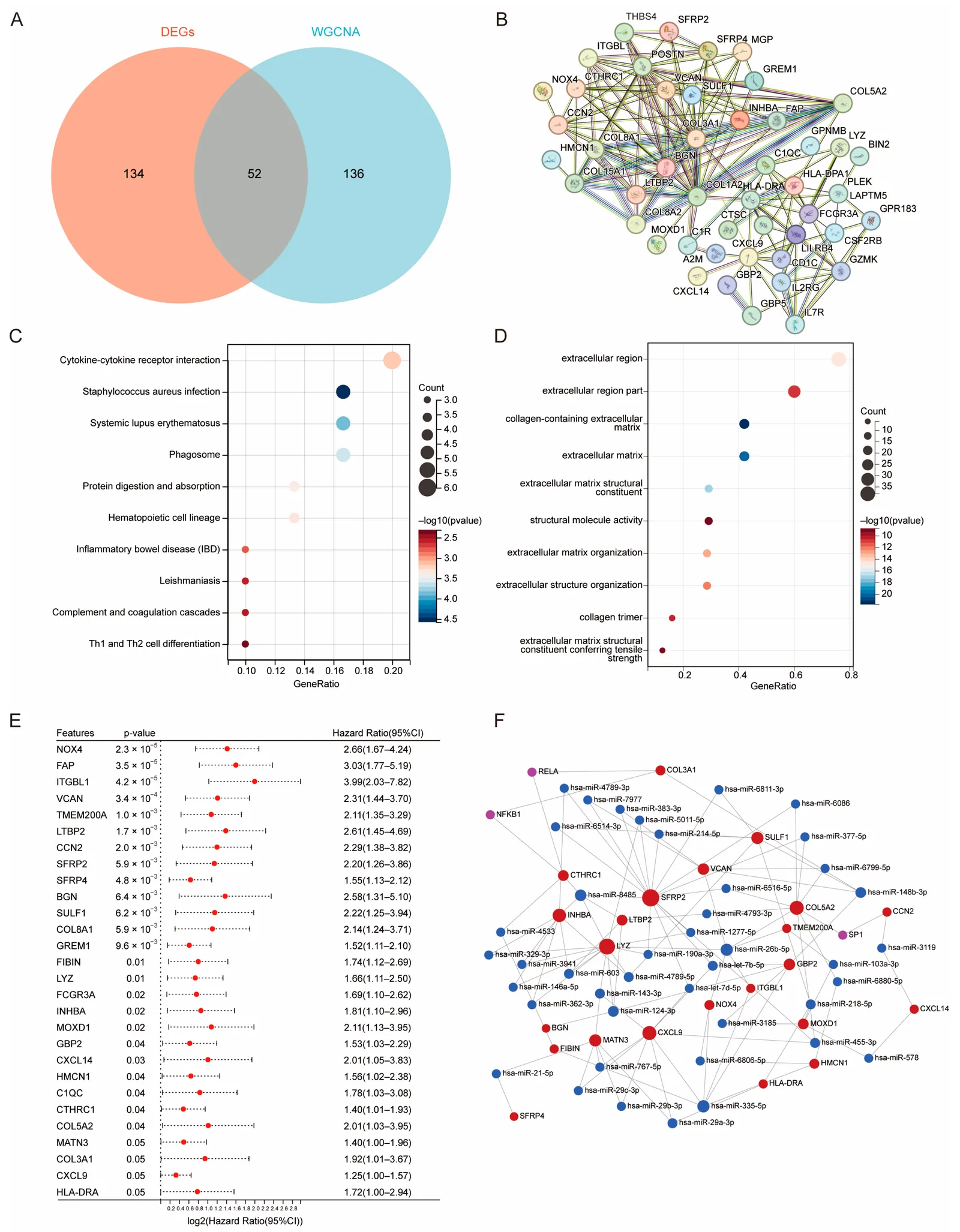
Selection and functional annotation of prognostic hub genes. (**A**) Venn diagram illustrating the overlap between DEGs and genes within the WGCNA black and red modules. (**B**) PPI network of the 52 intersecting hub genes. (**C**) Bubble plot of the top significantly enriched KEGG pathways among the 52 hub genes. (**D**) Bubble plot of the top significantly enriched GO terms for the 52 hub genes. (**E**) Forest plot of the 28 hub genes significantly associated with prognosis by univariate Cox regression. (**F**) Regulatory network depicting interactions between the 28 prognostic genes, their associated transcription factors (TFs), and microRNAs (miRNAs).

Fifty-two overlapping genes were identified at the intersection of C1-C2 DEGs and black/red WGCNA module genes (Figure 3A, Table S3) and defined as hub genes. A protein–protein interaction (PPI) network constructed from these hubs revealed core regulatory relationships (Figure 3B). KEGG pathway enrichment analysis showed significant enrichment in cytokine–cytokine receptor interaction, Staphylococcus aureus infection, and phagosome, etc. (Figure 3C). GO functional annotation linked these genes to the extracellular region, collagen-containing extracellular matrix, and related biological processes (Figure 3D). Univariate Cox regression of the 52 hubs identified 28 genes with significant prostate cancer prognostic correlation (Figure 3E). To explore post-transcriptional regulation, we built an integrated transcription factor–miRNA regulatory network (Figure 3F).

**Figure 4:**
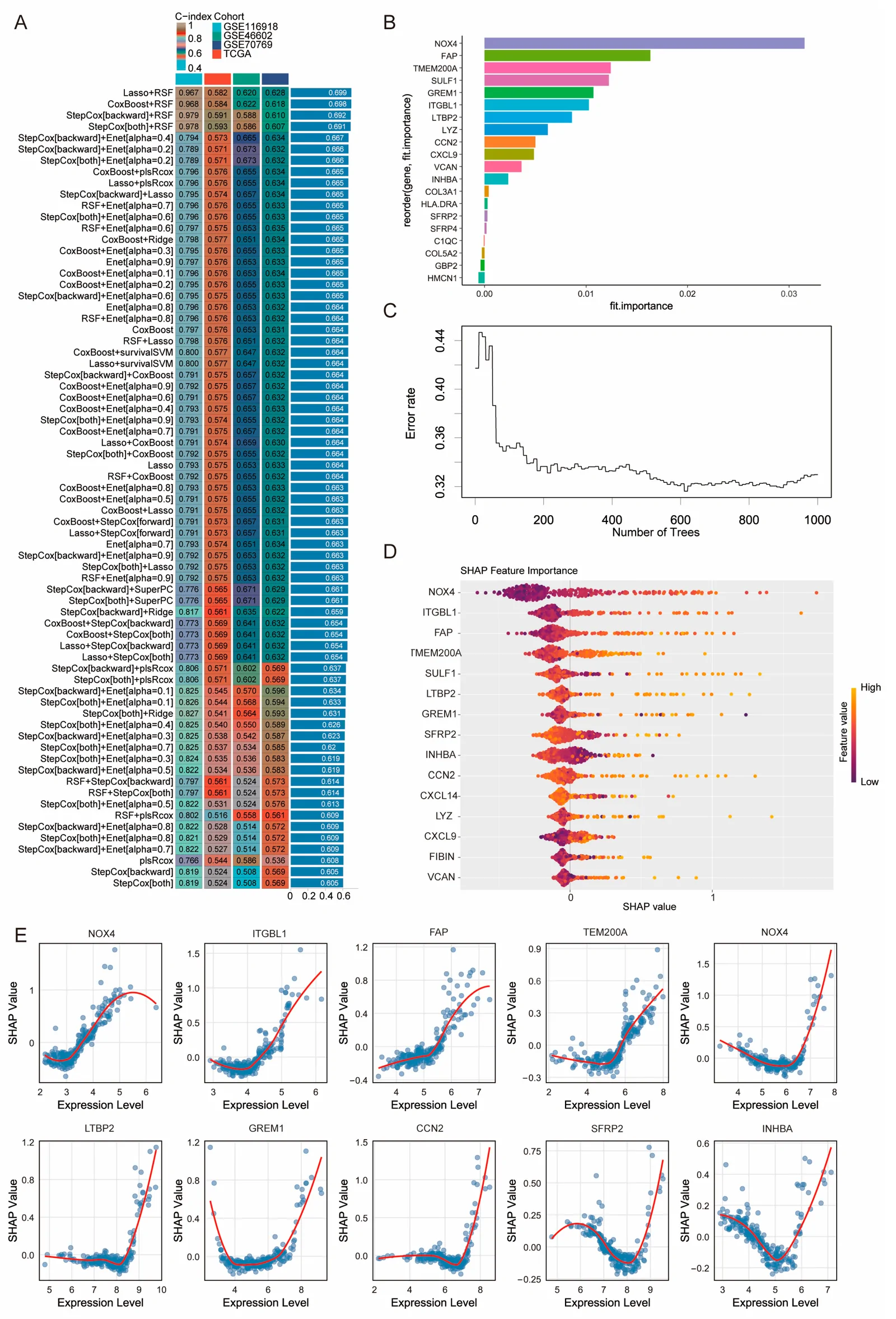
Machine learning-based prognostic model evaluation and interpretation. (**A**) Heatmap comparing the C-index of 91 machine learning models across the training and validation cohorts. (**B**) Feature importance ranking of the 28 genes as determined by the Random Forest algorithm. (**C**) Error rate plot of the Random Forest model across the number of trees. (**D**) SHAP bee swarm plot showing the mean absolute SHAP value for the top 15 most influential genes. (**E**) SHAP dependence plots for the top 8 genes, illustrating the relationship between gene expression and the model’s output value. Each blue dot represents an individual patient sample and the red fitted line (LOESS smoothing) summarizes the overall trend.

The “Mime1” package was used to construct 91 machine learning models evaluating the 28 prognostic genes’ predictive power. Derived from 9 basic algorithms, Random Forest-based models achieved the highest Harrell’s concordance index (C-index) in the GSE116918 training cohort and cross-cohort consistency evaluation cohorts (TCGA-PRAD, GSE46602, GSE70769) (Figure 4A). Feature importance rankings and Random Forest error rates are shown in Figure 4B,C. SHAP analysis improved model interpretability: a bee swarm plot displayed the 15 most predictive genes (Figure 4D), and dependence plots illustrated the top 8 gene expression effects on model output (Figure 4E).

**Figure 5:**
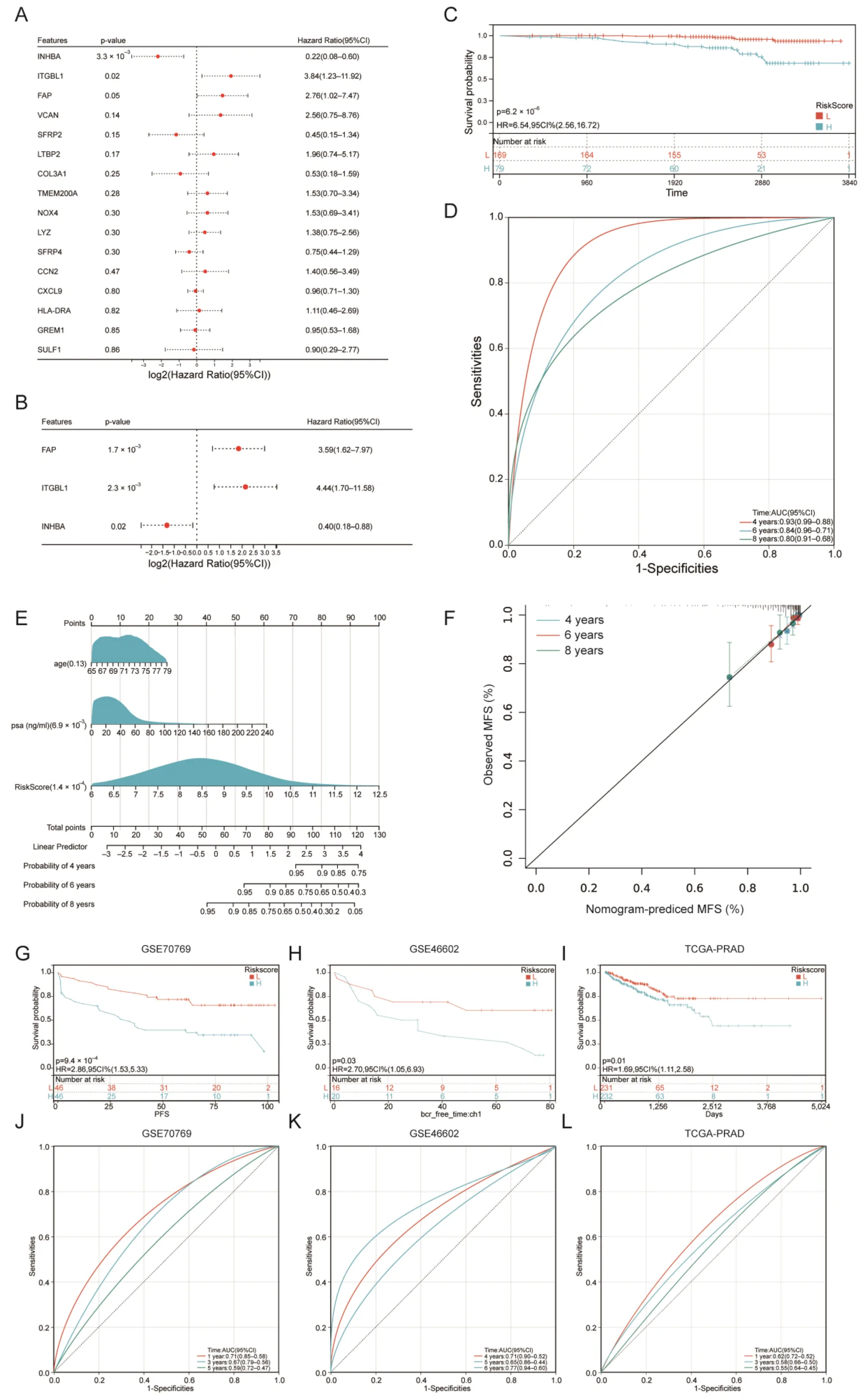
Construction and validation of a three-gene prognostic risk model. (**A**) Results of multivariable Cox regression analysis performed on the 16 genes with positive importance scores. (**B**) Forest plot of the final three genes retained in the multivariable Cox proportional hazards model. (**C**) Kaplan–Meier analysis of MFS between high-risk and low-risk groups in the training cohort. (**D**) Time-dependent ROC curves estimating the predictive accuracy of the risk score for 4-, 6-, and 8-year MFS in the training cohort. (**E**) A nomogram integrating age, the risk score, and PSA level to predict the probability of 4-, 6-, and 8-year MFS. (**F**) Calibration curve of the nomogram, comparing predicted versus observed MFS probability. (**G**–**I**) Kaplan–Meier survival analysis validating the risk model in three independent cohorts: (**G**) GSE70769, (**H**) GSE46602, and (**I**) TCGA-PRAD. (**J**–**L**) Time-dependent ROC analysis in the validation cohorts, (**J**) GSE70769, (**K**) GSE46602, and (**L**) TCGA-PRAD, showing the AUC at specified time points.

We performed multivariate Cox regression analysis on the 16 genes with positive feature importance identified by the Random Forest algorithm (Figure 5A), and three genes meeting the statistical significance threshold of *p* < 0.05 were selected to construct the final prostate cancer prognostic signature (Figure 5B). The coefficients used for building the model can be found in Table S4. A risk score was calculated for each patient based on the regression coefficients of the three genes from the multivariate Cox model and their respective expression levels. Patients were stratified into high-risk and low-risk groups according to the median risk score of the training cohort. Kaplan–Meier analysis confirmed that the high-risk group had significantly worse metastasis-free survival (MFS) than the low-risk group (Figure 5C). Time-dependent ROC curve analysis demonstrated the predictive accuracy of the three-gene signature, with AUC values of 0.93, 0.84, and 0.80 for predicting 4-year, 6-year, and 8-year MFS, respectively (Figure 5D). We further developed a nomogram integrating the three-gene risk score and pre-treatment PSA level, which was constructed as an exploratory training-cohort nomogram to predict the individual probability of 4-year, 6-year, and 8-year MFS in prostate cancer patients (Figure 5E). Calibration plots showed a high degree of consistency between the MFS probabilities predicted by the nomogram and the actual observed outcomes (Figure 5F). The DCA revealed that our model has certain clinical benefits compared to a single PSA in the training cohort (Figure S3). The prognostic performance of the risk score was evaluated in three cross-cohort consistency cohorts (GSE70769, GSE46602, TCGA-PRAD), and the high-risk group consistently showed directionally consistent associations with PFS and BCR in three cross-cohort consistency cohorts (Figure 5G–I). Time-dependent ROC analysis in these validation cohorts yielded maximum AUC values of 0.71 at 1 year for GSE70769, 0.77 at 6 years for GSE46602, and 0.62 at 1 year for TCGA-PRAD (Figure 5J–L).

**Figure 6:**
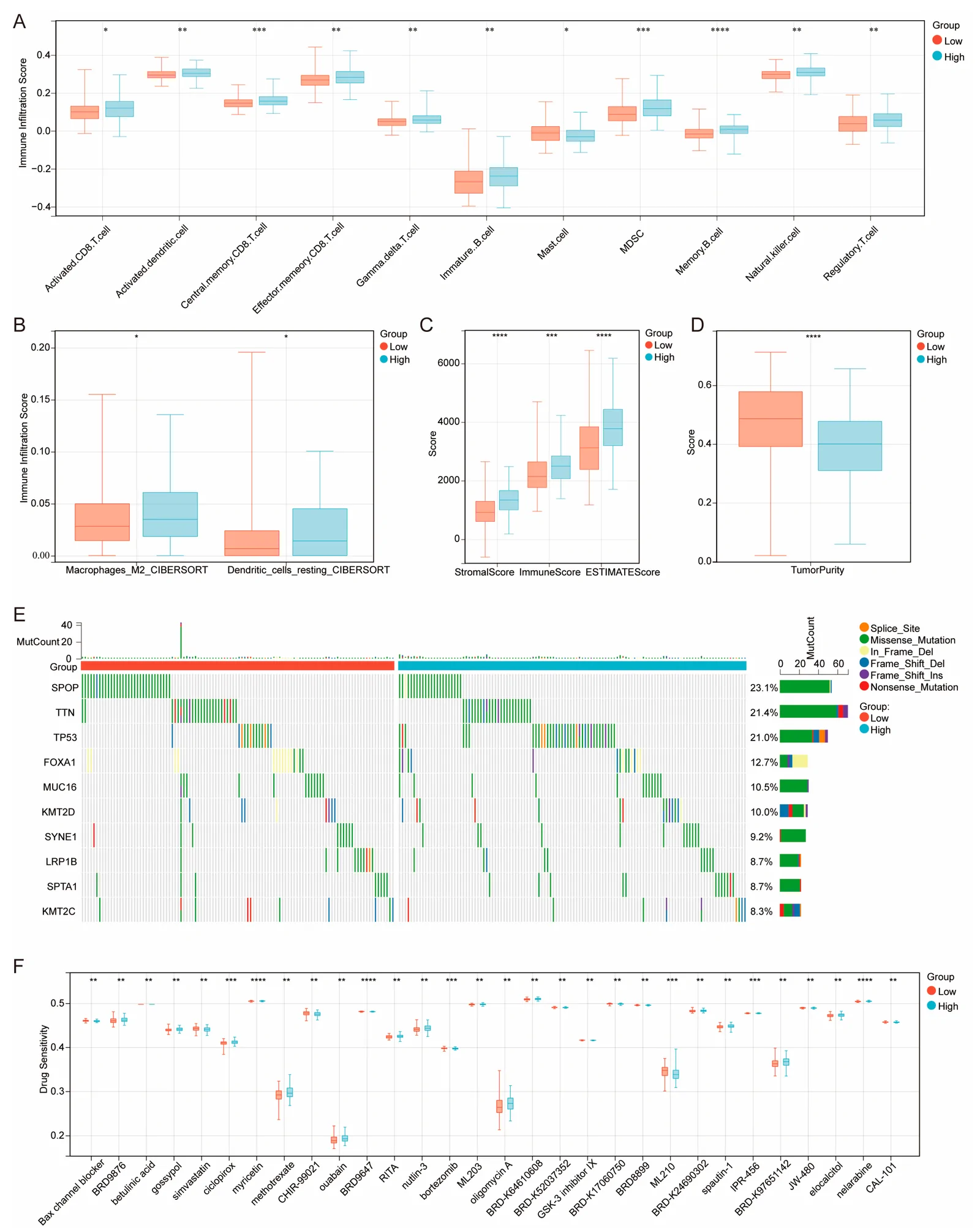
Comparative analysis of tumor microenvironment, mutational profile, and drug sensitivity between risk subgroups. (**A**) Box plots comparing the infiltration scores of various immune cell types between high-risk and low-risk groups, as estimated by multiple algorithms, * *p* < 0.05, ** *p* < 0.01, *** *p* < 0.001, **** *p* < 0.0001. (**B**) Comparison of M2 macrophage infiltration levels between subgroups using CIBERSORT, * *p* < 0.05. (**C**,**D**) Analysis of tumor microenvironment properties using the ESTIMATE algorithm (*** *p* < 0.001, **** *p* < 0.0001): (**C**) stromal, immune, and ESTIMATE scores and (**D**) tumor purity. (**E**) Oncoplot illustrating the mutation landscape of the top 10 most frequently mutated genes in the TCGA-PRAD cohort, stratified by risk subgroup. (**F**) Drug sensitivity prediction analysis, showing differential responses to selected therapeutic agents between high-risk and low-risk subgroups (** *p* < 0.01, *** *p* < 0.001, **** *p* < 0.0001).

The bulk transcriptomic analyses provided in silico estimates suggesting that the high-risk subgroup was enriched for activated CD8^+^ T cells, memory CD8^+^ T cells, dendritic cells, myeloid-derived suppressor cells (MDSCs), and regulatory T cells (Tregs) (Figure 6A). CIBERSORT analysis inferred that the infiltration level of M2-type macrophages was significantly elevated in the high-risk group (Figure 6B). Results from the ESTIMATE algorithm showed that the low-risk group had a higher tumor purity (Figure 6C) and lower StromalScore, ImmuneScore, and overall ESTIMATEScore values compared with the high-risk group (Figure 6D). These findings indicate that although the infiltration of immune cells in the high-risk subgroup is more significant, the population of immunosuppressive cells is also enriched in the high-risk group. This might be the key reason for the poor prognosis of these patients. We have presented a possible hypothesis here, but our proposals still require in-depth experiments for verification. Mutation analysis of the top 10 frequently mutated genes in the TCGA-PRAD cohort revealed that missense mutations were the main mutation type in both risk subgroups, while in-frame deletions were the predominant mutation type of FOXA1 in the low-risk group (Figure 6E). Drug sensitivity prediction analysis indicated that the high-risk group was more sensitive to chemotherapeutic drugs such as gossypol, ciclopirox, and methotrexate, whereas the low-risk group showed increased sensitivity to simvastatin, CHIR-99021, and ML210 (Figure 6F).

**Figure 7:**
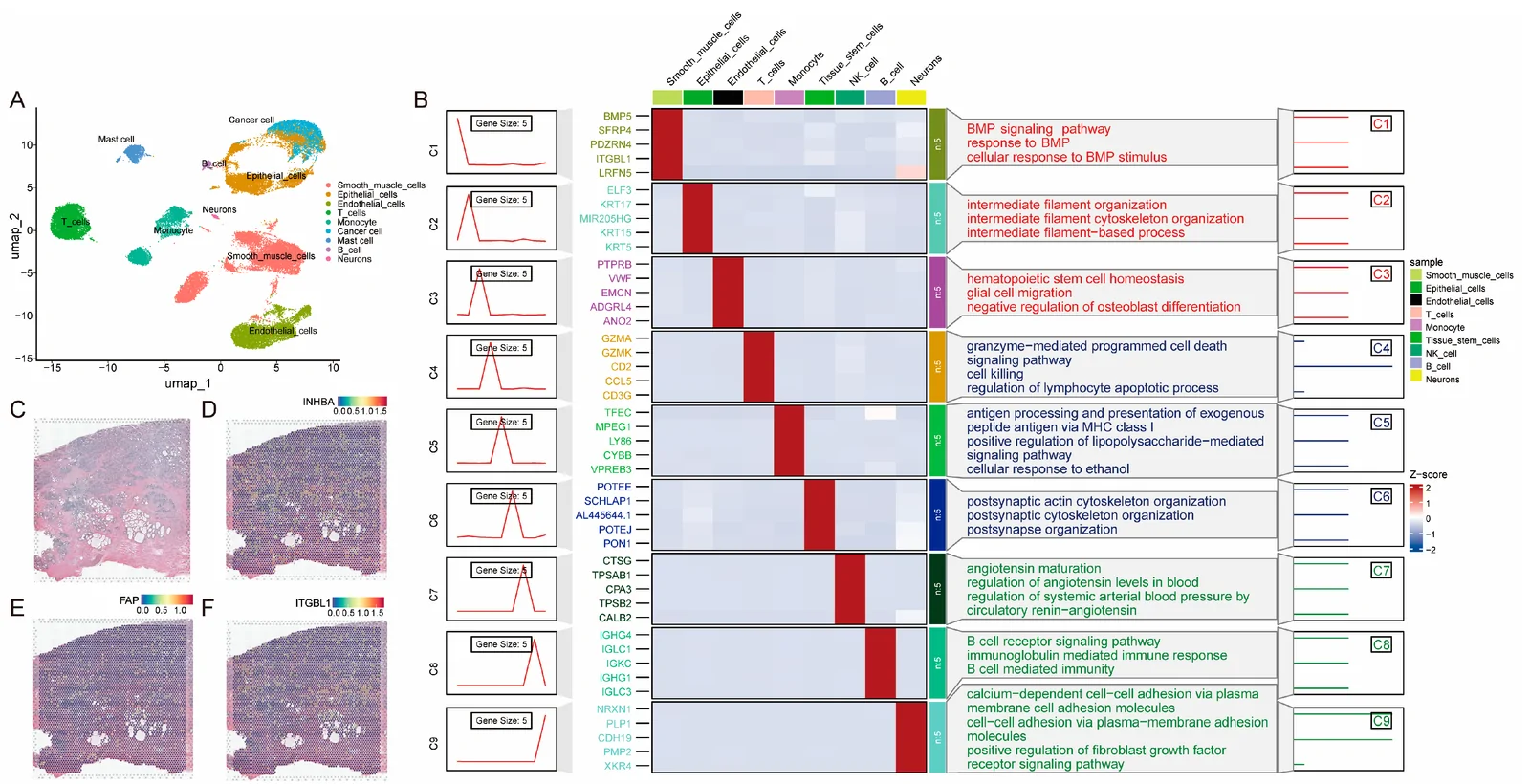
Single-cell and spatial transcriptomics reveal the localization of three hub genes. (**A**) UMAP projection of single-cell RNA sequencing data of two paired primary and metastatic prostate cancer samples, annotated by major cell types. (**B**) Heatmap of top marker genes and associated GO term clustering for the annotated cell types. (**C**) High-resolution H&E-stained image of the prostate cancer tissue section used for spatial transcriptomic profiling. (**D**–**F**) Spatial feature plots showing the expression patterns of the three hub genes: (**D**) INHBA, (**E**) FAP, and (**F**) ITGBL1.

We performed single-cell RNA sequencing (scRNA-seq) analysis on metastatic prostate cancer samples (GSE268307) and identified the major cell populations in the tumor microenvironment, including cancer cells, epithelial cells, fibroblasts, and various immune cells (Figure 7A). A heatmap of the top marker genes of each cell type combined with GO term clustering analysis suggested the distinct transcriptional characteristics of the annotated cell populations (Figure 7B). We also conducted manual marker annotations to further verify the results shown in Figure S4. To further resolve the spatial interaction of these cell types, we analyzed high-resolution spatial transcriptomics data of a human prostate adenocarcinoma sample. The tissue morphological structure was shown in Figure 7C. Spatial expression mapping of the three hub genes in the prognostic signature revealed their distinct spatial localization patterns (Figure 7D–F). We also calculated the density maps of three genes and found that they were enriched in smooth muscle cells (Figure S5A).

**Figure 8:**
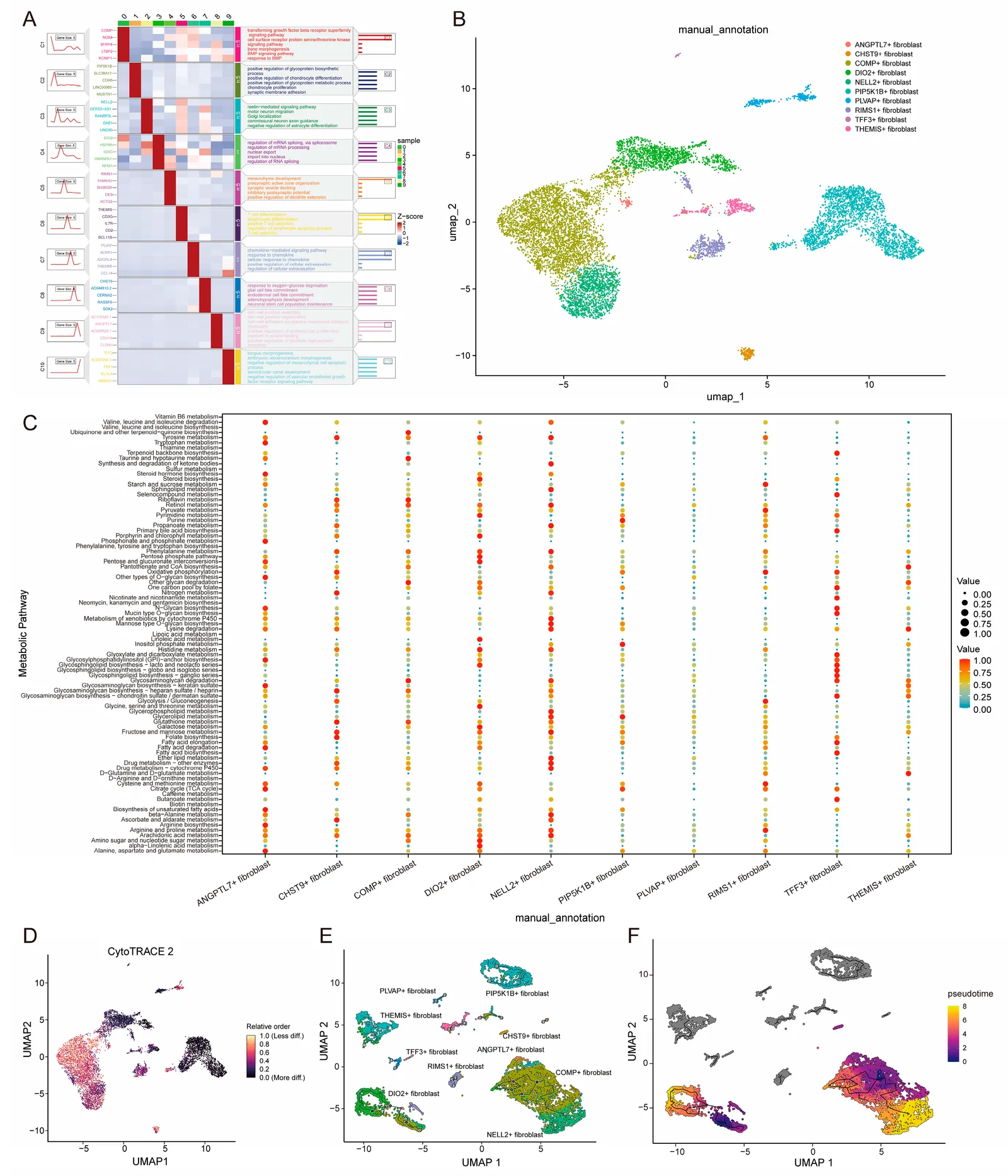
Developmental trajectory and metabolic profile of fibroblast subtypes. (**A**) Heatmap of top marker genes and GO term clustering for fibroblast subtypes identified after re-clustering of fibroblasts from scRNA-seq data. (**B**) UMAP visualization of fibroblast subpopulations. (**C**) Dot plot from scMetabolism analysis revealing distinct metabolic activities across fibroblast subtypes. (**D**) CytoTRACE prediction map overlaid on the fibroblast UMAP, indicating high differentiation potential for the COMP+ subset. (**E**) Cells ordered along a pseudotime axis, colored by fibroblast subcell type. (**F**) Cell density distribution along the inferred pseudotime, illustrating a differentiation path from COMP+ to NELL2+ fibroblasts.

**Figure 9:**
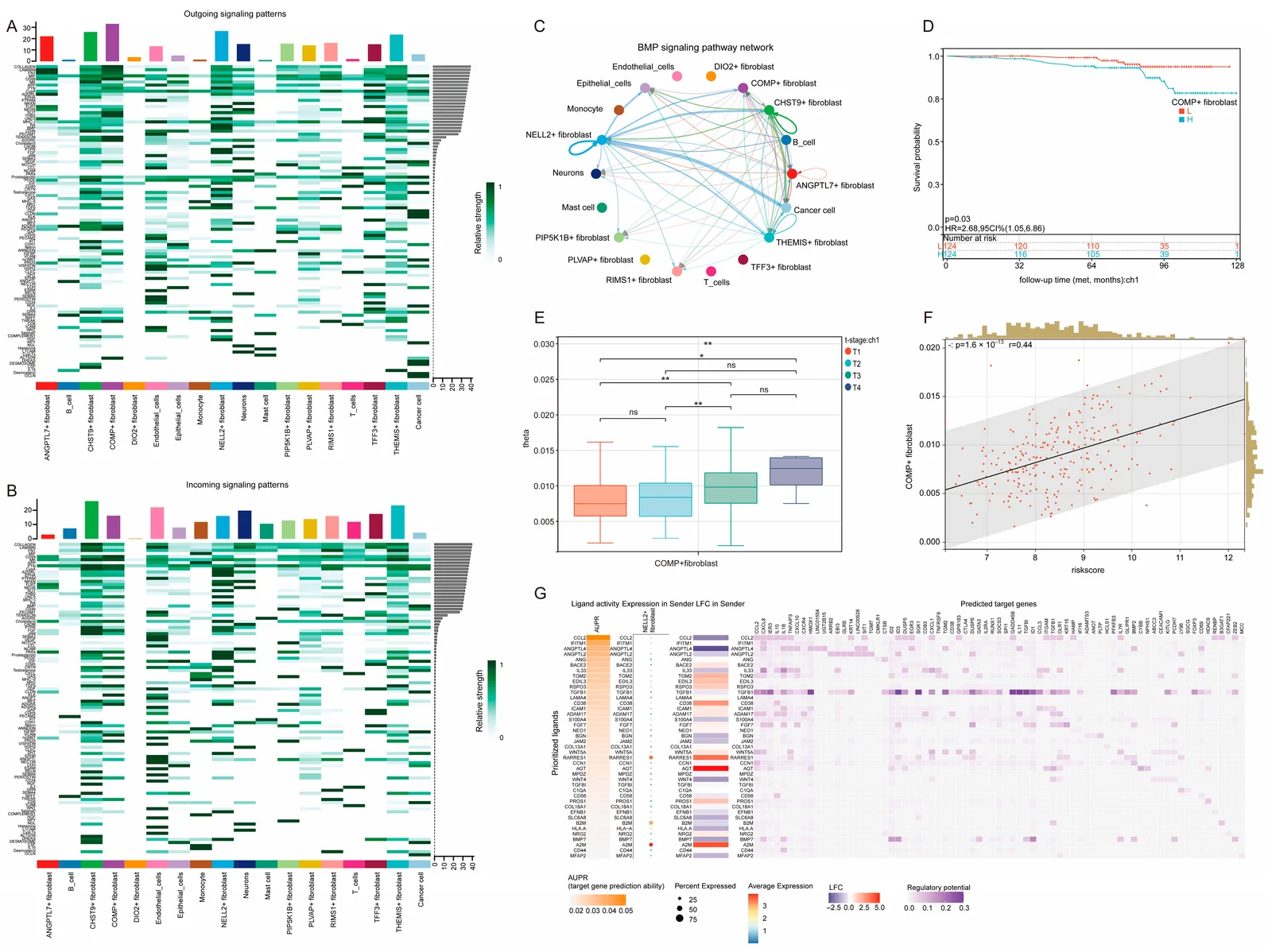
NELL2+ fibroblast-driven BMP signaling and its clinical translational significance. (**A**,**B**) Heatmaps of cell–cell communication analysis at the single-cell level: (**A**) Outgoing interaction strength pattern and (**B**) incoming interaction strength pattern, highlighting potent BMP signaling from NELL2+ fibroblasts to cancer cells. (**C**) Circos plot detailing the specific ligand–receptor interactions constituting the BMP signaling pathway between NELL2+ fibroblasts and cancer cells. (**D**) Kaplan–Meier curve demonstrating significantly worse metastasis-free survival for patients with high abundance of COMP+ fibroblasts, as inferred by BayesPrism deconvolution of bulk transcriptomic data. (**E**) Box plot showing a significant increase in COMP+ fibroblast abundance with advancing pathological T stage in the GSE116918 cohort, * *p* < 0.05, ** *p* < 0.01, ns stands for *p* > 0.05. (**F**) Scatter plot showing a positive correlation between the prognostic risk score and the inferred abundance of COMP+ fibroblasts. Each blue dot represents an individual patient sample. The red fitted line (linear regression) and the shaded band (95% confidence interval) illustrate the overall trend and the uncertainty of the fit, further supporting the association between COMP^+^ fibroblast infiltration and adverse prognosis in prostate cancer. (**G**) Heatmap from NicheNet analysis prioritizing ligands from COMP+ fibroblasts that regulate pro-tumorigenic target genes in receiver cells.

We further dissected the heterogeneity of fibroblasts in the prostate tumor microenvironment by re-clustering the fibroblast population from the scRNA-seq data, which identified several fibroblast subtypes, including COMP+ fibroblasts and NELL2+ fibroblasts; the subtyping results and marker gene expression were visualized by UMAP and heatmap combined with GO analysis (Figure 8A,B). Three hub genes were all enriched in the COMP+ fibroblast (Figure S5B). Metabolic profiling analysis via the scMetabolism package revealed distinct metabolic characteristics among these fibroblast subtypes (Figure 8C). The differentiation potential estimation conducted using CytoTRACE indicated that COMP+ fibroblasts possess a relatively high degree of differentiation ability (Figure 8D). Pseudotemporal trajectory analysis constructed with the Monocle3 package indicated a continuous differentiation trajectory from COMP+ fibroblasts to NELL2+ fibroblasts based on calculations (Figure 8E,F). Based on this developmental hierarchy, we analyzed the outgoing and incoming intercellular communication signals of different fibroblast subtypes. Interaction strength analysis revealed that NELL2+ fibroblasts are key signal senders of the BMP signaling pathway, and the target cells of this signaling are mainly cancer cells (Figure 9A,B). A circos plot was used to detail the specific ligand–receptor pairs that constitute the activated BMP signaling pathway (Figure 9C). The spatial slices obtained through RCTD deconvolution revealed the localization of COMP+ fibroblasts, NELL2+ fibroblasts and cancer cells in the prostate tumor microenvironment (Figure S6A–C). In addition, AUCell scoring of the BMP signaling pathway (based on MSigDB gene sets) and spatial expression analysis of key BMP pathway members (BMP7, BMPR1B) revealed that BMP signaling activity was significantly elevated in tissue regions rich in COMP+ and NELL2+ fibroblasts (Figure S6D–F).

BayesPrism deconvolution of prostate cancer bulk transcriptomic data suggested that patients with high inferred COMP+ fibroblast abundance had significantly worse metastasis-free survival (Figure 9D). COMP+ fibroblast abundance increased with T stage progression (Figure 9E) and positively correlated with the three-gene signature’s prognostic risk score (Figure 9F). NicheNet analysis inferred upstream regulatory ligands secreted by COMP+ fibroblasts, prioritizing key ligands that may regulate hub genes and other pro-tumorigenic targets in recipient cells (Figure 9G). The functional enrichment of BMP7 (a key BMP pathway ligand) downstream targets showed significant enrichment in TGF-β signaling and stem cell pluripotency pathways (Figure S7), providing a computational hypothesis linking fibroblast-driven BMP signaling to aggressive cancer cell phenotypes.

## 4. Discussion

Prostate cancer (PCa) remains the most frequently diagnosed malignancy and the second leading cause of cancer-related mortality among men worldwide [31]. Despite advancements in clinical management that have contributed to a gradual decline in mortality rates, the annual incidence of PCa continues to rise, underscoring an urgent need for more effective prognostic tools and therapeutic strategies [32]. Epithelial–mesenchymal transition (EMT) represents a critical developmental process that is co-opted during carcinogenesis, conferring enhanced invasive and metastatic properties upon tumor cells [33,34,35]. This cellular reprogramming, characterized by loss of epithelial features and acquisition of a mesenchymal phenotype, is governed by multiple signaling pathways, including PI3K/Akt, TGF-β/Smad, and Wnt/β-catenin [36,37,38]. Our machine learning-derived three-gene prognostic model effectively stratified patients into subgroups with significantly distinct clinical outcomes. High-risk patients exhibited elevated levels of immunosuppressive cells—including regulatory T cells, M2-polarized macrophages, and myeloid-derived suppressor cells—alongside reduced tumor purity. Recent machine-learning-based imaging and grading tools, such as Telecan et al. [39], have shown utility for ISUP grade prediction in prostate cancer. These imaging-based approaches complement molecular risk-stratification strategies. Integrating molecular signatures with imaging features may further improve prostate cancer risk assessment, although such integration requires prospective validation. These findings suggest that the tumor microenvironment may evade immune surveillance despite a heightened overall immune presence, representing a paradoxical state that potentially explains the aggressive clinical course in this subgroup. Beyond the prognostic model, our single-cell and spatial transcriptomic analyses revealed previously unappreciated cellular heterogeneity within the prostate tumor microenvironment. We identified a novel COMP+ fibroblasts subpopulation with high differentiation potential, which gave rise to NELL2+ fibroblasts through a distinct developmental trajectory through bioinformatics analysis. These fibroblast subsets are likely to play roles in shaping the tumor ecosystem: COMP+ fibroblasts demonstrated significant association with adverse prognosis and advanced disease stage, while NELL2+ fibroblasts established potent BMP signaling toward cancer cells, potentially driving tumor progression. Spatial deconvolution and ligand–receptor interaction analysis also revealed the interactions between fibroblasts and tumor cells, which to some extent supported the results of our previous analysis. Regarding the individual genes in our signature, INHBA, a member of the TGF β superfamily, encodes the beta A subunit of activin and inhibin [40]. Homodimerization produces activin A, while heterodimerization with the inhibin alpha subunit results in inhibin A [41]. The INHBA in our model seems to be somewhat contradictory. It was a risk factor in the single-factor analysis but a protective factor in the multi-factor analysis. We conducted an in-depth analysis of this phenomenon in the Supplementary Materials (Figure S8) and believe that INHBA may exhibit different effects depending on the level of expression, rather than being the result of multicollinearity. This seems to be valuable for future research. Previous research indicates that activin A can inhibit the growth of breast and ovarian epithelial cells via Smad activation and CDK inhibitor induction [42], while other studies suggest INHBA modulates cell growth and apoptosis through the PI3K/AKT pathway [43]. The mechanisms through which FAP and ITGBL1 contribute to immunosuppression and metastasis warrant further investigation. FAP encodes fibroblasts activation protein, expressed on reactive stromal fibroblasts in epithelial tumors [44]. TGF-β1 regulates stromal fibroblast-mediated EMT in bladder cancer via the FAP/VCAN axis, where FAP upregulation promotes CAF-like activation [45]. Our single-cell analysis revealed fibroblast-specific FAP expression, consistent with its potential role in prostate cancer progression. ITGBL1, encoding integrin beta-like protein 1, promotes metastasis in various cancers, including breast and ovarian malignancies [46,47,48], though it exhibits tissue-specific functions as evidenced by its suppressive role in non-small cell lung cancer [49]. In PCa, ITGBL1 appears to activate NF-κB signaling to promote invasion and migration, consistent with our findings [50]. In summary, this study demonstrates the utility of integrating multi-omics data and computational algorithms to identify novel biomarkers. We identified three EMT-related genes—INHBA, FAP, and ITGBL1—and developed a new three-gene signature for prognostic stratification in PCa, providing a foundation for targeting EMT and immune evasion pathways. Several limitations should be acknowledged: (1) The training cohort was limited to 248 patients treated with radical radiotherapy, which was small relative to the 91 machine-learning models evaluated and potentially introduced selection bias toward the Random Forest–Cox model. No surgery- or ADT-treated cohorts were used for training. The substantial decrease from the training C-index (0.93) to external validation C-indices (0.62–0.77) suggests possible cohort-specific overfitting or unaccounted clinical heterogeneity. In addition, the model was trained for MFS but evaluated using PFS in TCGA-PRAD and BCR in GSE70769 and GSE46602. These endpoints capture different clinical events and cannot be considered equivalent to MFS. Therefore, the external results provide only exploratory cross-cohort evidence and do not constitute independent validation of the MFS model. In this case, it should be regarded as exploratory rather than clinically applicable. (2) Lack of experimental validation: Although we have made many bold speculations in bioinformatics, ultimately, biological experiments are still needed to validate them. We will consider conducting more in-depth research based on this study in the future. (3) Potential confounders: Factors such as lifestyle, comorbidities, and medications were not adjusted for and may influence gene expression and prognostic associations. (4) Lack of single-cell and spatial transcriptomics samples: The 4 single-cell samples and 1 spatial transcriptomics sample used for analysis may introduce bias. (5) A key limitation is that only FAP among the final three genes remained significant after FDR correction, indicating that the training-cohort-only feature selection was permissive and may be prone to overfitting. In addition, some downstream analyses involved repeated comparisons without comprehensive correction, confidence intervals, or effect sizes.

## 5. Conclusions

Our study develops and validates a novel three-gene prognostic signature (INHBA, FAP, ITGBL1), providing new insights into predicting the survival free from metastasis or biochemical recurrence. This signature identifies an aggressive subtype characterized by EMT activation and a highly immunosuppressive tumor microenvironment. High-resolution spatial and single-cell transcriptomics revealed that these three genes were enriched in a novel developmental-specific COMP+ fibroblast subpopulation. Our bioinformatics speculation suggested that COMP+ fibroblasts may have strong differentiation potential and could differentiate into NELL2+ fibroblasts, and activate a potent BMP signaling pathway in cancer cells, thereby promoting tumor progression/metastasis. BayesPrism deconvolution found that COMP+ fibroblasts had a positive correlation with poor prognosis and advanced stage. In conclusion, our study provides a new exploratory prognostic model for prostate cancer risk stratification and offers a new biological information-based explanation for potential prostate cancer metastasis and invasion: targeting the signaling pathways driven by COMP+ fibroblasts to reverse immunosuppression and inhibit metastasis and spread.

## Data Availability

The data supporting the findings of this study are openly available in public repositories. The bulk transcriptomic data used for model training are available from the Gene Expression Omnibus (GEO) under accession number GSE116918 (https://www.ncbi.nlm.nih.gov/geo/query/acc.cgi?acc=GSE116918 (accessed on 10 July 2025)). Validation bulk data are available from GEO under accessions GSE70769 (https://www.ncbi.nlm.nih.gov/geo/query/acc.cgi?acc=GSE70769 (accessed on 7 August 2025)), GSE46602 (https://www.ncbi.nlm.nih.gov/geo/query/acc.cgi?acc=GSE46602 (accessed on 7 August 2025)), and from The Cancer Genome Atlas Prostate Adenocarcinoma (TCGA-PRAD) project (https://portal.gdc.cancer.gov/ (accessed on 20 June 2025)). The single-cell RNA sequencing data are available from GEO under accession GSE268307 (https://www.ncbi.nlm.nih.gov/geo/query/acc.cgi?acc=GSE268307 (accessed on 5 August 2025)). The spatial transcriptomics data are available from the 10x Genomics public database (https://www.10xgenomics.com/datasets/human-prostate-cancer-adenocarcinoma-with-invasive-carcinoma-ffpe-1-standard-1-3-0 (accessed on 22 July 2025)). Epithelial–mesenchymal transition (EMT)-related gene sets were obtained from MSigDB (https://www.gsea-msigdb.org/gsea/msigdb (accessed on 20 June 2025)) and dbEMT (https://bioinfo-minzhao.org/dbemt/dbemt1/index.html (accessed on 20 June 2025)). All other data supporting the findings of this study are available within the article and its Supplementary Materials or from the corresponding author upon reasonable request.

## Supplementary Materials

The following supporting information can be downloaded at: https://www.mdpi.com/article/10.3390/genes17091015/s1, Figure S1: Supplementary figures related to EMT gene analysis and survival; Figure S2: Identification of differentially expressed genes and co-expression modules associated with the aggressive cluster (C2); Figure S3: The DCA analysis revealed that the nomogram model provided preliminary internal evidence compared to a single PSA or age measurement in the GSE116918; Figure S4: The density UMAP map used for manual marker annotation to verify the correctness of the main cell clusters; Figure S5: UMAP plot showing the density distribution of three hub genes in all the celltype (A) and only in the fibroblasts (B); Figure S6: Illustrative spatial analysis from one tissue section of RCTD results and BMP pathway members; Figure S7: KEGG pathway enrichment analysis for genes predicted to be regulated by BMP7, showing significant enrichment in TGF-β signaling and stem cell pluripotency pathways; Figure S8: The detailed analysis of the opposite effect of INHBA; Table S1: EMT-related gene sets; Table S2: Univariate analysis; Table S3: VennData; Table S4: coef of three genes.

## Abbreviations

PCa	Prostate Cancer
EMT	Epithelial–Mesenchymal Transition
ADT	Androgen Deprivation Therapy
AUC	Area Under the Curve
BCR	Biochemical Recurrence
BFS	Biochemical Recurrence-Free Survival
CI	Confidence Interval
C-index	Concordance Index
DEG	Differentially Expressed Gene
CDF	Cumulative distribution function
FAP	fibroblasts Activation Protein
FDR	False Discovery Rate
GEO	Gene Expression Omnibus
GO	Gene Ontology
GSVA	Gene Set Variation Analysis
GWAS	Genome-Wide Association Study
HR	Hazard Ratio
INHBA	Inhibin Subunit Beta A
ITGBL1	Integrin Subunit Beta Like 1
KEGG	Kyoto Encyclopedia of Genes and Genomes
KDM	Klemera-Doubal Method
MFS	Metastasis-Free Survival
OR	Odds Ratio
PCA	Principal Component Analysis
PPI	Protein–Protein Interaction
PSA	Prostate-Specific Antigen
RCTD	Robust Cell Type Decomposition
ROC	Receiver Operating Characteristic
SHAP	SHapley Additive exPlanations
SNP	Single-Nucleotide Polymorphism
ssGSEA	Single-Sample Gene Set Enrichment Analysis
TCGA	The Cancer Genome Atlas
TCGA-PRAD	The Cancer Genome Atlas Prostate Adenocarcinoma
TF	Transcription Factor
TMB	Tumor Mutational Burden
TOM	Topological Overlap Matrix
WGCNA	Weighted Gene Co-Expression Network Analysis
LOOCV	leave-one-out cross-validation
MDSCs	Myeloid-derived Suppressor Cells
