# Supplementary material for: Integrating Multi-Omics and Machine Learning to Reveal a Prognostic Model for Prostate Cancer Metastatic Recurrence Associated with Epithelial–Mesenchymal Transition Features
Source: Genes (Basel). 2026 Aug 27;17(9):1015. doi: 10.3390/genes17091015 (PMC13606394; doi:10.3390/genes17091015)
Supplement: Supplementary file 1 [file genes-17-01015-s001.zip › genes-4460834-supplementary/Supplementary Figures.pdf]

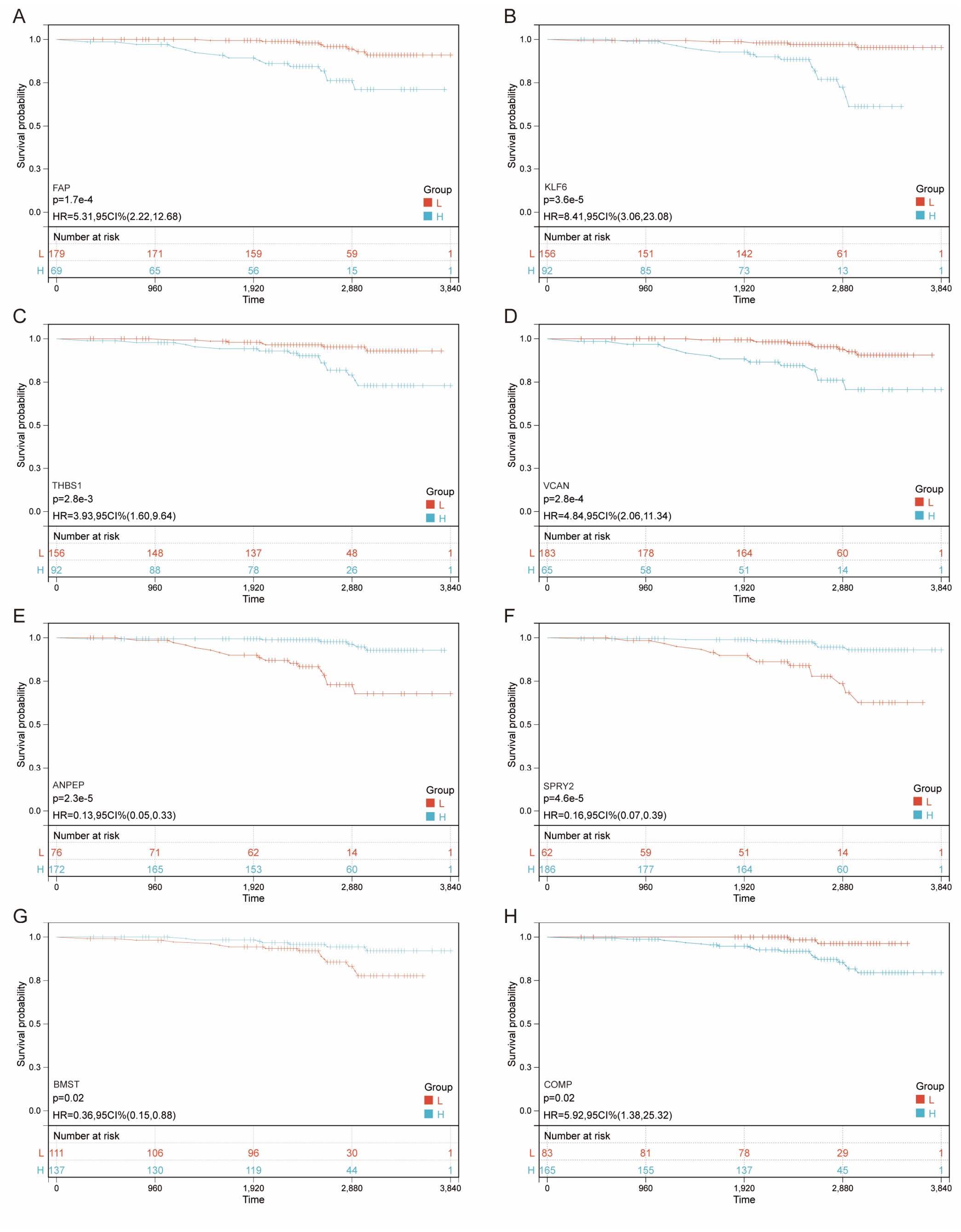

Figure S1

Supplementary figures related to EMT gene analysis and survival.

(A-H) Kaplan-Meier survival curves for the top 8 individual EMT-related genes with the smallest P-values from univariate Cox analysis.

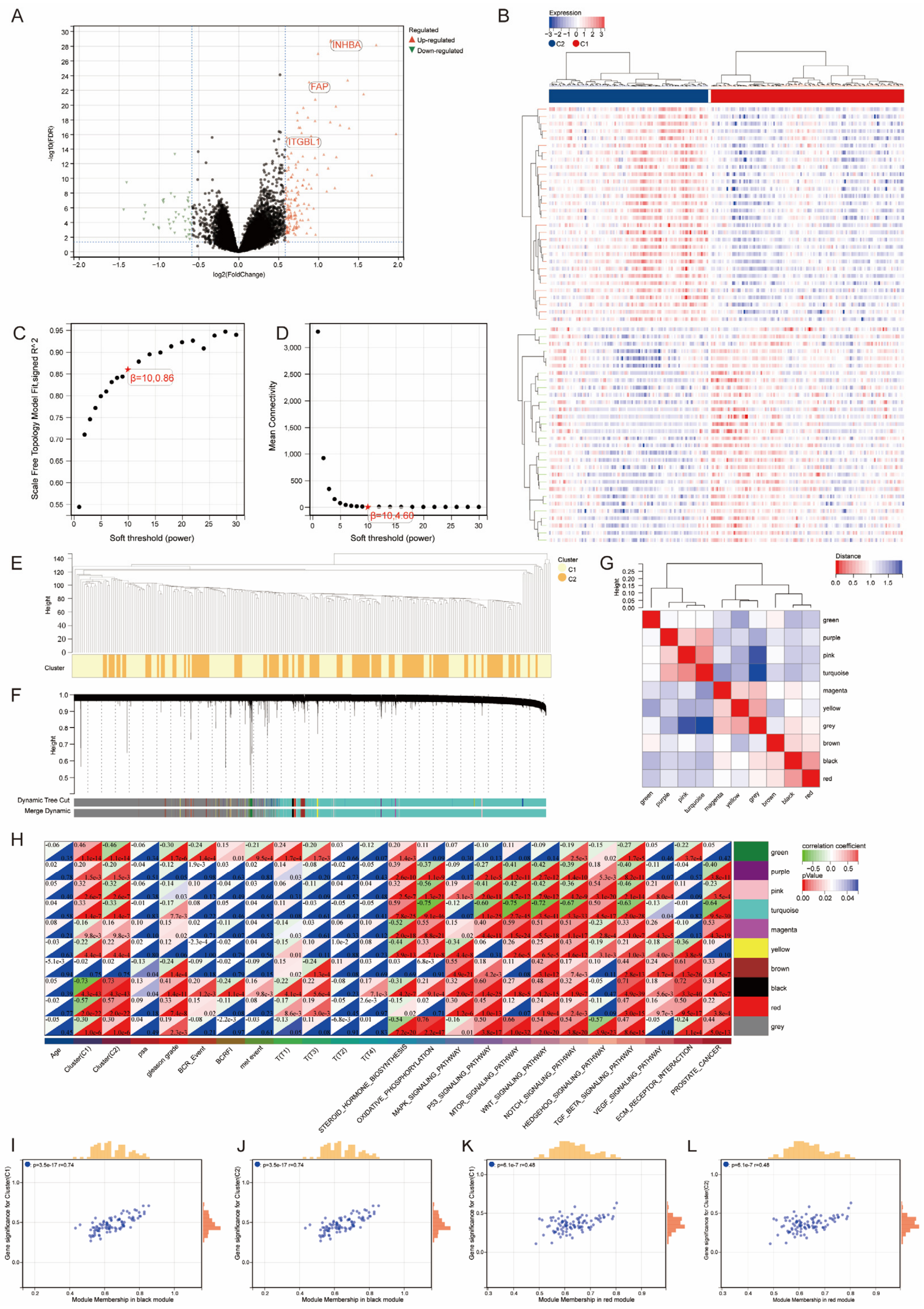

Figure S2

Identification of differentially expressed genes and co-expression modules associated with the aggressive cluster (C2).

(A) Volcano plot of DEGs between clusters C2 and C1.

(B) Heatmap showing the expression patterns of the top 30 upregulated and top 30 downregulated genes in C2 versus C1.

(C-D) Analysis of network topology for various soft-thresholding powers (β) to achieve scale-free topology in WGCNA. (C) Scale independence. (D) Mean connectivity.
(E) Sample clustering dendrogram and trait heatmap.
(F) Gene clustering dendrogram with assigned module colors.
(G-H) Heatmaps of module-trait relationships correlating gene modules with (G) the cluster phenotype and (H) pathway activity scores.
(I-L) Scatterplots of module eigengene values versus the cluster phenotype for the (I-J) black and (K-L) red modules.

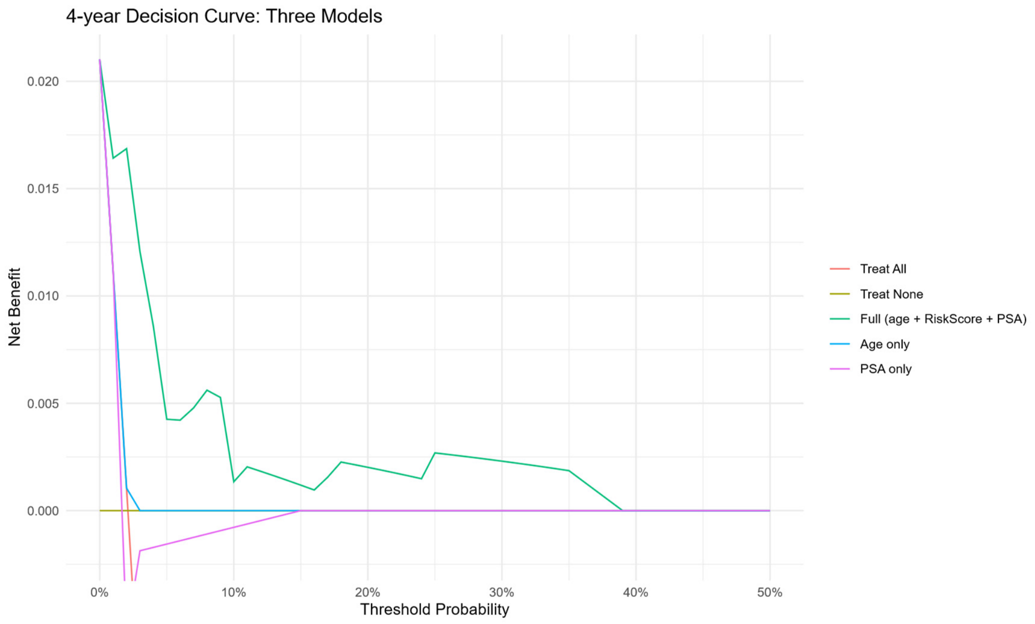

Figure S3

The DCA analysis revealed that the nomogram model provided preliminary internal evidence compared to a single PSA or age measurement in the GSE116918.

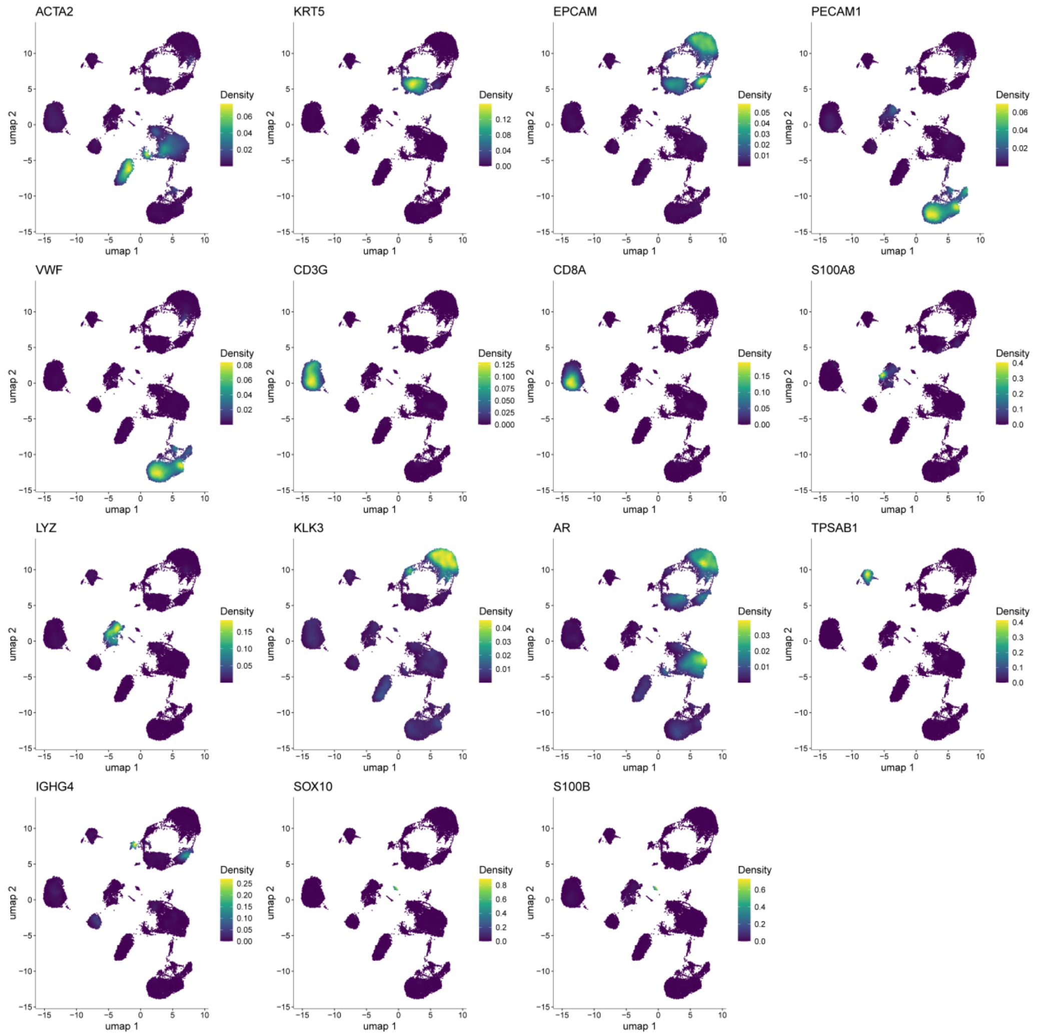

Figure S4

The density UMAP map used for manual marker annotation to verify the correctness of the main cell clusters. ACTA2 for smooth muscle cells, KRT5 and EPCAM for epithelial cells, PECAM1 and VWF for endothelial cells, CD3G and CD8A for T cells, S100A8 and LYZ for monocytes, KLK3 and AR for cancer cells, TPSAB1 for mast cells, and IGHG4 for B cells, SOX10 and S100B for neurons.

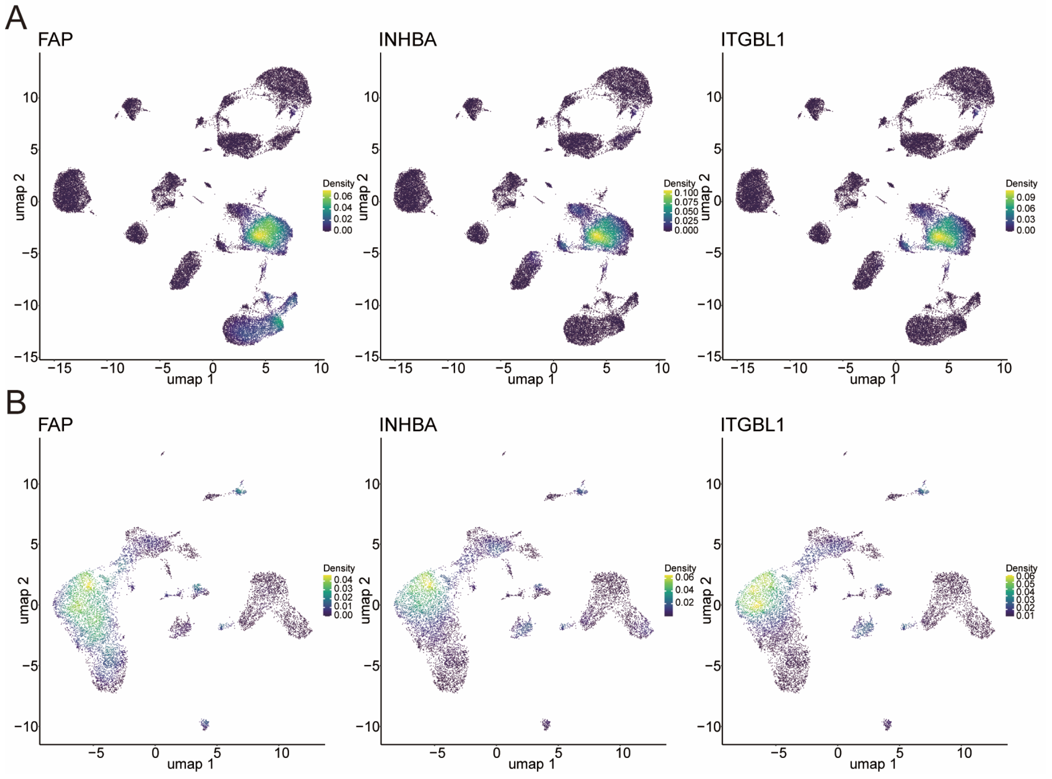

Figure S5

UMAP plot showing the density distribution of three hub genes in all the celltype (A) and only in the fibroblasts (B)

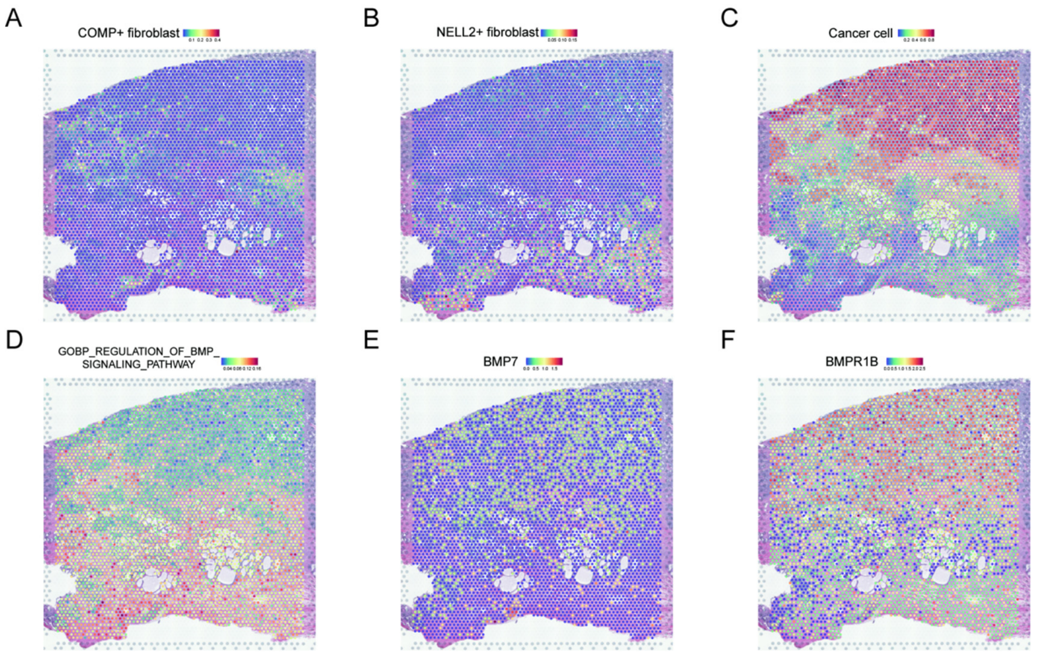

Figure S6

Illustrative spatial analysis from one tissue section of RCTD results and BMP pathway members.

(A-C) Spatial distribution of cell types estimated by RCTD deconvolution: (A) COMP+ fibroblasts, (B) NELL2+ fibroblasts, (C) Cancer cell.
(D) Spatial visualization of the BMP pathway activity score calculated by AUCell using gene sets from MSigDB.
(E, F) Spatial feature plots showing the expression of key BMP pathway members (E) BMP7 and (F) BMPR1B.

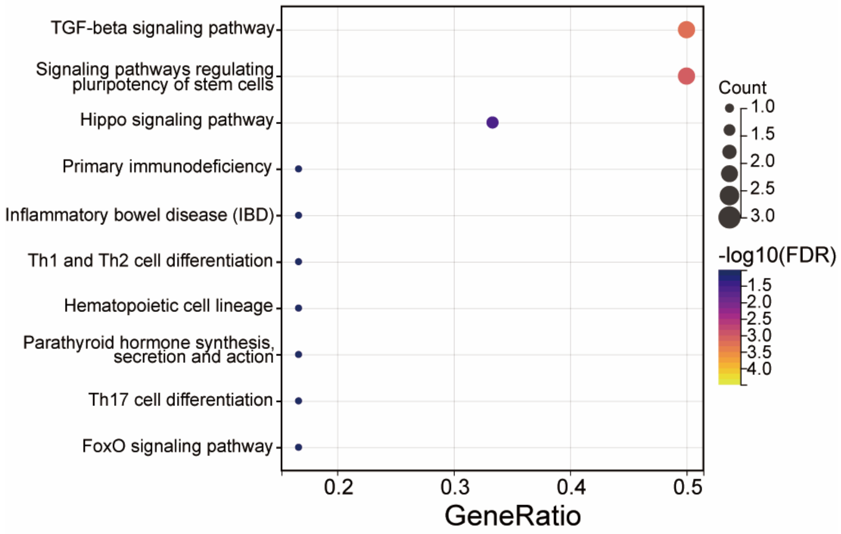

Figure S7 **KEGG pathway enrichment analysis for genes predicted to be regulated by BMP7, showing significant enrichment in TGF-β signaling and stem cell pluripotency pathways.**

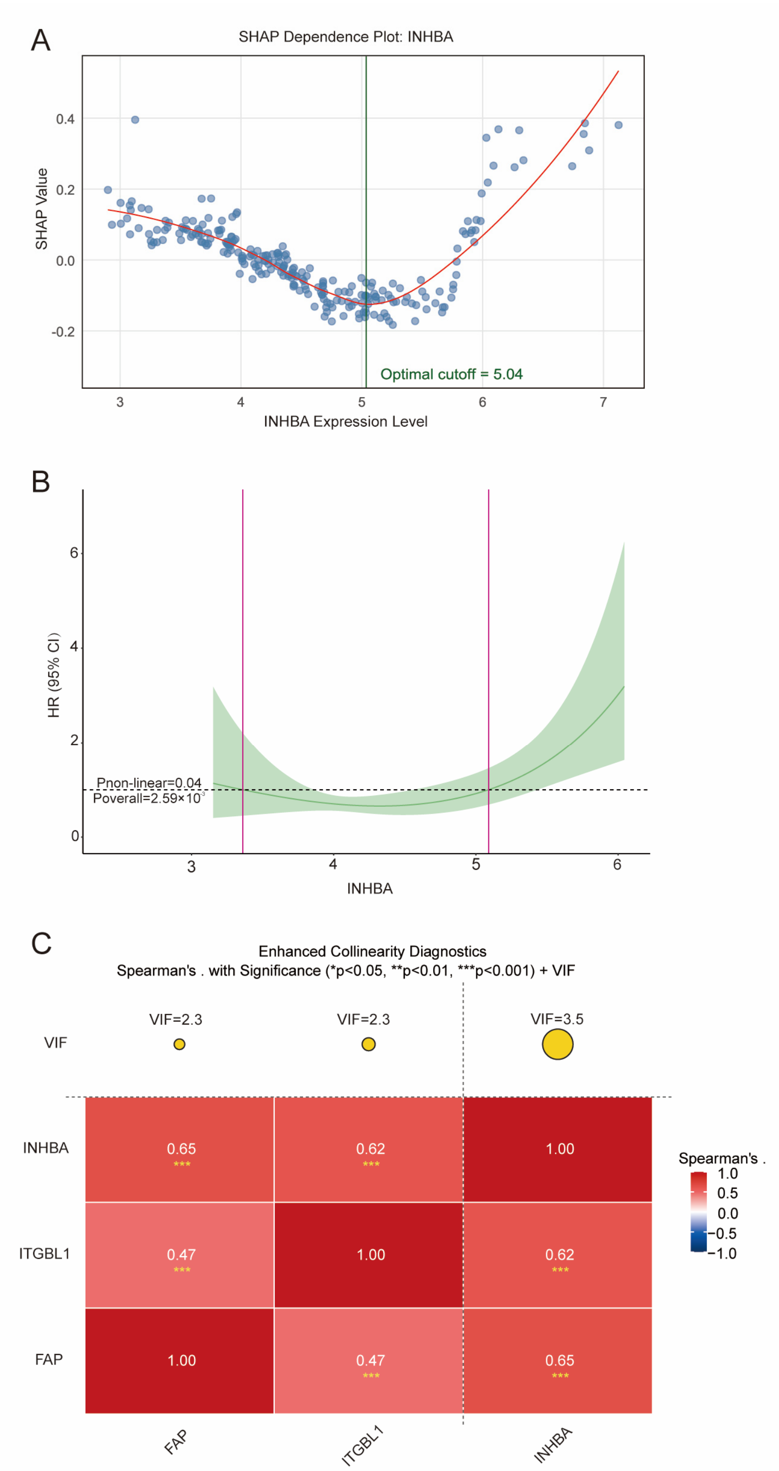

Figure S8 the detailed analysis of the opposite effect of INHBA.

(A) The SHAP analysis revealed that its contribution exhibited a U-shaped effect. (B) The RCS analysis revealed that INHBA has a non-linear effect on HR. (C) The heatmap reveals the correlations of the three genes and their VIF values.
